# Reduced infant rhesus macaque growth rates due to environmental enteric dysfunction and association with histopathology in the large intestine

**DOI:** 10.1038/s41467-021-27925-x

**Published:** 2022-01-11

**Authors:** Sara M. Hendrickson, Archana Thomas, Kamm Prongay, Andrew J. Haertel, Laura M. Garzel, Leanne Gill, Tasha Barr, Nicholas S. Rhoades, Rachel Reader, Mark Galan, Julie M. Carroll, Charles T. Roberts, Lina Gao, Ian J. Amanna, Ilhem Messaoudi, Mark K. Slifka

**Affiliations:** 1 Division of Neuroscience, Oregon National Primate Research Center, Oregon Health & Science University, Beaverton, OR 97006 USA; 2 Division of Comparative Medicine, Oregon National Primate Research Center, Oregon Health & Science University, Beaverton, OR USA; 3 California National Primate Research Center, University of California, Davis, Davis, CA USA; 4 Department of Molecular Biology and Biochemistry, University of California Irvine, Irvine, CA USA; 5 Department of Pathology and Laboratory Medicine, Rutgers, New Jersey Medical School, Newark, NJ USA; 6 Division of Cardiometabolic Health and Division of Reproductive and Developmental Science, Oregon National Primate Research Center, Oregon Health & Science University, Beaverton, OR 97006 USA; 7 Biostatistics and Bioinformatics Core, Oregon National Primate Research Center, and Biostatistics Shared Resource, Knight Cancer Institute, Portland, OR 97239 USA; 8 Najít Technologies, Inc., Beaverton, OR 97006 USA

**Keywords:** Intestinal diseases, Gastrointestinal system, Infectious diseases

## Abstract

Environmental enteric dysfunction is associated with malnutrition as well as infant growth stunting and has been classically defined by villous blunting, decreased crypt-to-villus ratio, and inflammation in the small intestine. Here, we characterized environmental enteric dysfunction among infant rhesus macaques that are naturally exposed to enteric pathogens commonly linked to human growth stunting. Remarkably, despite villous atrophy and histological abnormalities observed in the small intestine, poor growth trajectories and low serum tryptophan levels were correlated with increased histopathology in the large intestine. This work provides insight into the mechanisms underlying this disease and indicates that the large intestine may be an important target for therapeutic intervention.

## Abstract (web summary)

Environmental enteric dysfunction (EED), a subclinical disorder of intestinal function, is associated with malnutrition, infant growth stunting and pathologies in the small intestine. Here the authors report that infant rhesus macaques that are naturally exposed to enteric pathogens commonly linked to human growth stunting present with EED and growth faltering that associates with large intestine pathologies.

## Introduction

Globally, approximately one-quarter of children under 5 years of age are growth-stunted^1^. The first few years of life are critical for both physical and cognitive development^2^ but by 2 years of age, nearly 40% of children in Peru and up to 71% of children in Tanzania are growth-stunted^3^. Although the frequency of early infant diarrheal episodes have been associated with significantly reduced growth trajectories^4^, this is not always a consistent finding^5^. Growth stunting is also observed in the absence of overt diarrhea and is believed to be due to environmental enteric dysfunction (EED)^6^, a disease associated with subclinical enteric pathogen infection^7^ (particularly *Campylobacter* species^8–10^), increased gut inflammation, and reduced growth trajectories^6,11–15^.

The central hypothesis of EED is that repeated enteric infections contribute to undernutrition by causing intestinal inflammation and alterations in absorptive functions as well as the intestinal barrier^7^. Here, we found that infant rhesus macaques are naturally exposed to a number of enteric pathogens known to be associated with EED and growth stunting. These animals presented with a phenotype consistent with EED, including the development of considerable villous atrophy and other histological abnormalities of the small intestine that were observed prior to 1 year of age. However, when stratified according to growth rates, histological abnormalities in the small intestine were identified equally among both healthy and slow-growing infant rhesus macaques. In contrast, animals with the poorest growth trajectories had a significantly higher incidence of histopathological lesions in the large intestine. This finding was further supported by an inverse correlation between serum tryptophan levels and histopathology in the large intestine, indicating a link between poor nutrient absorption and reduced infant growth trajectories. Together, these results show that EED is not strictly a disease of the small intestine and suggests that the colon/large intestine may become an important energy salvage organ when nutrient absorption in the small intestine is compromised. This indicates that interventions that target both large and small intestines may be needed for effective treatment and prevention of EED-associated growth/cognitive stunting among at-risk children.

## Results

### Enteric pathogen exposure

**Fig. 1:**
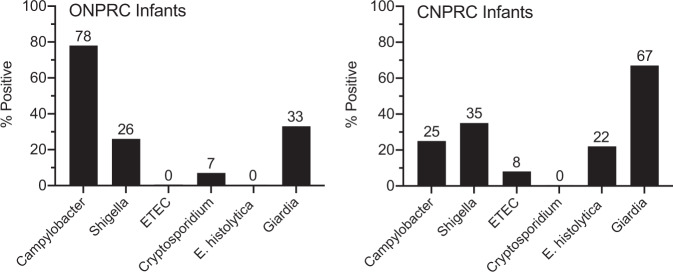
Natural infection of outdoor-housed rhesus macaques with human enteric pathogens. Rhesus macaques were screened for enteric pathogens associated with human infant growth stunting at 1 month of age with 40 infants tested at each primate center. The numbers in the panels indicate the percentage positive results for each pathogen based on direct microbial culture (*Campylobacter*) or by xTAG PCR (*Shigella*, ETEC; Enterotoxigenic *E. coli*, *Cryptosporidium*, *E. histolytica*, and *Giardia*). ONPRC Oregon National Primate Research Center, CNPRC California National Primate Research Center. Source data are provided as a Source Data file.

We examined naturally occurring enteric disease among outdoor-housed rhesus macaques at the Oregon National Primate Research Center (ONPRC) and the California National Primate Research Center (CNPRC)^16–19^. The animals are housed in family groups of 25–100 individuals with access to clean water and balanced nutrition. Young monkeys are curious by nature and engage in hand-to-mouth exploration and although the housing floor substrates are flushed daily, fecal waste may accumulate between cleanings and lead to early-age exposure to a number of enteric pathogens. This explorative behavior, coupled with these exposure conditions, appears to model some of the challenges associated with implementing water, sanitation, and hygiene (WASH) interventions among young children in developing countries^20^. Interestingly, prior studies indicate that the gut microbiome of rhesus macaques is clearly distinct from that of human cohorts from the United States and instead more closely resembles the microbiomes observed in developing countries and resource-poor settings such as Malawi, Burkina Faso, Bangladeshi slums, and Amerindians from Venezuela^21–23^. Using direct microbial culture screening and a multiplex PCR-based diagnostic assay (xTAG^24–26^), we determined the incidence of a subset of enteric pathogens among infant rhesus macaques at the ONPRC and CNPRC, including parasites such as Giardia^27^ that would not have been identified previously by bacterial 16 S rRNA microbiome analysis (Fig. 1). Several pathogens identified among the rhesus macaques are associated with human growth stunting and include *Campylobacter*^8–10,28–30^, *Shigella*^29,31^, enterotoxigenic *Escherichia coli* (ETEC)^28,29^, *Cryptosporidium*^29,31^, *Entamoeba histolytica*^32,33^, and *Giardia*^29,34^. Although diarrheal disease is relatively common among outdoor-housed non-human primates (NHP)^16–18,27^, none of the 1-month-old infants (*n* = 80) presented with clinical diarrhea at the time of screening for enteric pathogens. In terms of parasitic infections, subclinical *Giardia* was the most common and was identified among 33 to 67% of infants at the ONPRC and CNPRC, respectively. *Campylobacter* represented the most common bacterial pathogen associated with human growth stunting at the ONPRC, with 78% of infants colonized within 1 month after birth. *Campylobacter* was also identified among 25% of the infant macaques at the CNPRC and by 6 months of age, 100% of infant macaques followed longitudinally at either site had scored *Campylobacter-*positive by the microbial culture at one or more time points. *Shigella* was the most common EED-associated bacterial pathogen at the CNPRC with 35% of infants scoring positive compared to 26% of infants at ONPRC. Together, these data indicate that outdoor-housed rhesus macaques are exposed to a number of clinically relevant enteric pathogens at an early age, notably *Campylobacter*, *Shigella*, and *Giardia*.

### Histological evidence of EED among infant rhesus macaques

**Fig. 2:**
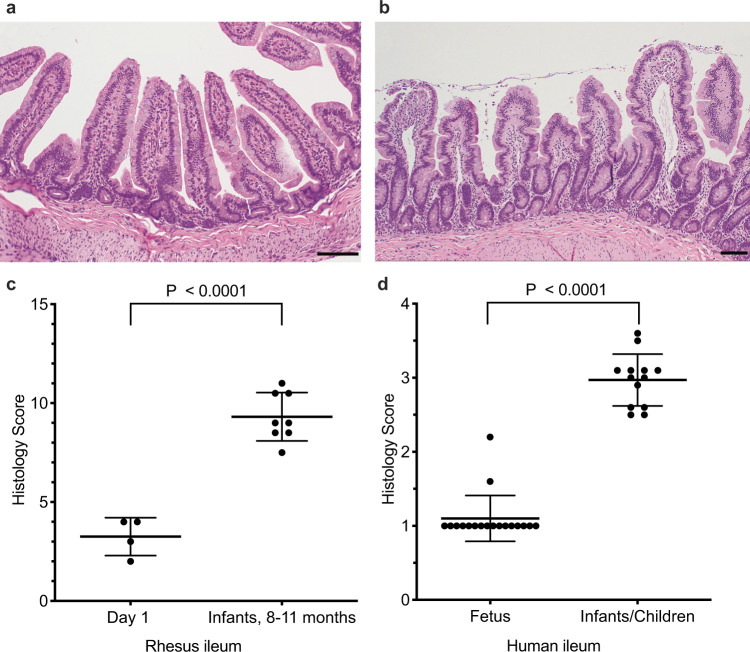
Histological analysis of environmental enteric dysfunction (EED) in rhesus macaques. EED is classically defined by histological abnormalities in the small intestine. To determine if villous architecture changes between birth and early infancy in the absence of clinically apparent diarrheal disease, hematoxylin and eosin-stained tissue samples from the distal ileum of rhesus macaques at 1 day of age (**a**) or 11 months of age (**b**) were examined. A 100 μM scale bar is included in each panel. **c** Tissue samples from animals at 1 day of age (*n* = 4) or 8–11 months of age with no history of clinical diarrhea (*n* = 8) were scored by two independent pathologists who were blinded to animal age or group designation (Mean±SD) using predefined histopathology criteria (Supplementary Table 1). **d** Histology scores (Mean ± SD) were calculated from the published work by Chacko et al.^37^ that described autopsy samples of terminal ileum obtained from the human fetus (*n* = 18) or infants/children who died of diseases other than those of the GI tract (*n* = 13). *P* values are based on unpaired student’s *t*-test. Source data are provided as a Source Data file.

**Fig. 3:**
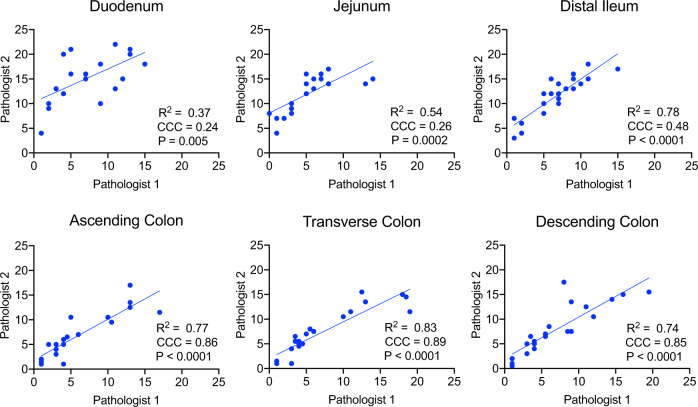
Independent assessment of blinded histology samples indicates reproducibility of scoring criteria. Hematoxylin and eosin-stained histology slides from each of the indicated anatomical locations were given a randomized identity code and assessed independently by two blinded pathologists. Scores were unblinded and graphed to show the relationship between scores provided by a veterinary pathologist (Pathologist 1) and a clinical pathologist (Pathologist 2). CCC Concordance Correlation Coefficients. *P* values were determined by univariable linear regression. Source data are provided as a Source Data file.

EED is believed to be an environmentally-acquired enteropathy of the small intestine, leading to inflammation, villus blunting, and decreased crypt-to-villus ratio^12,35^ along with other variable features that may indicate remodeling of the villus architecture. Although the majority of human histopathology has been focused on duodenal biopsies, to our knowledge there has not been a systematic analysis of the entire gastrointestinal (GI) tract among infants or small children diagnosed with EED. To determine if early-life encounters with enteric pathogens that are endemic among group-housed rhesus macaques would result in histological abnormalities that have been associated with EED, we selected animals with varying medical histories for histological analysis of the small intestine (Fig. 2 and Supplementary Fig. 1). Hematoxylin and eosin-stained tissue slides from each animal were given randomized identity codes that were scored independently in a blinded fashion by two pathologists (one veterinary pathologist who specializes in non-human primate pathology and has extensive experience in gastrointestinal pathology and one a clinical MD pathologist who specializes in gastrointestinal pathology) using pre-determined scoring criteria (Tables 1, 2 and Supplementary Tables 1, 2). Although the veterinary pathologist scored the blinded small intestine samples (duodenum, jejunum, and distal ileum) modestly lower than the clinical pathologist, this was not observed with large intestine samples (ascending colon, transverse colon, and descending colon) and in both cases, the trends in histology scores were highly correlated with each other. The duodenum showed the greatest variability (*R*^2^ = 0.37), but this is known within the field; different locations of duodenum tissue within a single slide may vary in terms of histological abnormalities, and depending on which area of a given section is evaluated, differences in scoring are not unexpected. Indeed, in a recent histology study of EED involving a pediatric Pakistani cohort^35^, the duodenum was described as “patchy” and when within-host duodenal biopsies were compared, there was a higher degree of discordance of overall histologic scores between samples from the same child than there were between those of different children enrolled in the same study. In terms of the rhesus macaque studies described here, the histopathology scores provided by each blinded pathologist were highly consistent (*P* ≤ 0.005, Fig. 3) and were averaged to provide final values.

**Table 1:** Criteria and assigned values for microscopic changes in the duodenum, jejunum, and ileum.

Value	0	1	2	3
Villus: crypt ratio	≥4:1	2:01	1:01	01:00.5
Villous blunting	Absent	Present in <25% of villi	Present in 25–50% of villi	Present in >50% of villi
Villous fusion	Absent	Present in <25% of villi	Present in 25–50% of villi	Present in >50% of villi
Number of intraepithelial lymphocytes	0–5	6–20	21–40	>40
Lymphoplasmacytic infiltrates	Normal background	Mild increase	Moderate increase	Severe increase
Eosinophilic infiltrates	Normal background	Mild increase	Moderate increase	Severe increase
Neutrophilic infiltrates	Normal background	Mild increase	Some clustering	Prominent clustering or eosinophilic cyrptitis
Inflammation of submucosa	Normal	Mild	Severe	
Macrophage clustering	Absent	Minimal	Prominent	
Enterocyte necrosis or apoptosis	Absent	Present

**Table 2:** Criteria and assigned values for microscopic changes in the cecum, colon, and rectum.

Value	0	1	2	3
Mucosal hyperplasia	None	Mild	Moderate	Severe
Presence of neutrophils	None	Mild	Moderate	Severe
Lymphoplasmacytic infiltrates	Normal background	Mild increase	Moderate increase	Severe increase
Crypt abscessation	None	Mild	Moderate	Severe
Surface epithelial tufting/dysplasia	None	Mild	Moderate	Severe
Presence of enteric parasites*	None observed	One type of organism	Two types of organisms	Three or more types of organisms
Absence of normal filamentous^+^	Bacteria present	Bacteria absent		
Intraepithelial lymphocytes	0–5	6–20	21–40	>40
Surface epithelial injury	None	Mild	Moderate or few microerosions	Severe or prominent microerosions
Paneth cells	Absent	Rare	Prominent	
Lamina propria macrophages	Absent	Minimal	Prominent	
Inflammation of submucosa	Normal	Mild	Severe	
Goblet cells	Present	Mildly reduced	Moderately reduced	

*Organisms observed were *Balantidium coli* (ciliated protozoan), *Trichuris* sp (nematode), flagellated protozoa within crypts, and uncultured long filamentous bacteria within crypts.

^+^Presence or absence of normal superficial filamentous bacteria were scored as 0 to 1 with segmental loss scored as 0.5.

Since infant macaques are colonized with a number of enteric pathogens (Fig. 1) and expected to have accrued histological abnormalities at an early age, we prepared histological sections from four animals necropsied at 1 day of age as negative controls and compared these to samples from eight animals at 8–11 months of age that had no history of acute or chronic diarrhea indicative of clinically apparent enterocolitis^16,17,27^ and no history of chronic diarrhea with or without abdominal bloating that might be indicative of celiac disease^36^ (Fig. 2). Similar to healthy human ileum samples^37^, the ileum of animals examined at 1 day of age showed long finger-like villi with a high villus:crypt ratio, rare or absent villous blunting or fusion, little to no inflammation of the submucosa, and normal background of eosinophilic/neutrophilic infiltrates (Fig. 2a). In contrast, clinically asymptomatic animals at 8–11 months of age showed histological lesions that are associated with EED, including substantial villous blunting, low villus:crypt ratios, and varying degrees of intestinal inflammation (Fig. 2b). There was a significant difference in histology scores (*P* < 0.0001) between these two groups of animals with none of the infants at 8–11 months of age demonstrating the pristine villous structures with lack of histological abnormalities observed among the day 1 samples (Fig. 2c). The 2.9-fold difference in the structural architecture of the macaque small intestine was similar to the 2.7-fold difference in histological scores that were observed in prior postmortem studies after autopsy of small intestine samples from stillborn human fetuses in comparison with infants and small children who had lived under the same endemic conditions but died of diseases unrelated to the GI tract^37^ (Fig. 2d). Histological abnormalities in the small intestine currently represents a key feature of EED^35,38^ and similar to humans, aberrations in villous architecture observed among infant macaques are not genetic but instead, appear to be due to environmental factors that cause abnormalities among the villi of the small intestine at an early age.

### Association between gut histopathology and infant growth faltering

**Fig. 4:**
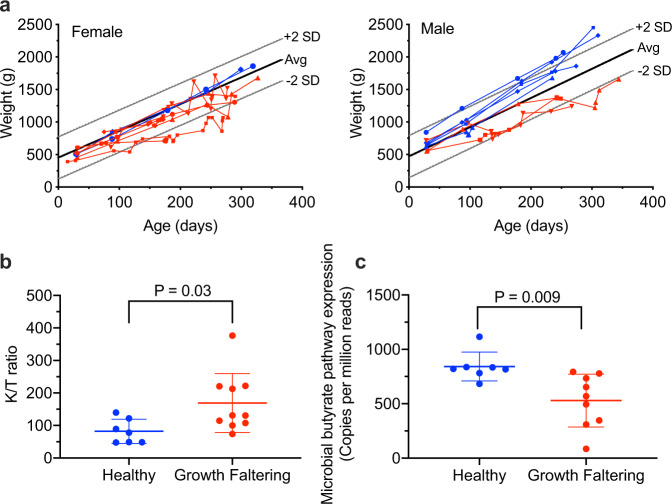
Growth rates of healthy and growth faltering infant rhesus macaques. The average (Avg) growth rate (bold lines) ±2 SD (standard deviation, thin lines) were prepared from published data on male (*n* = 719) and female (*n* = 781) rhesus macaques reared in standard outdoor sheltered housing^17^. **a** Individual growth rates of 17 infant macaques were graphed on the indicated sex-specific growth scale for females or males. Animals that grew faster than average were designated healthy (*n* = 7, blue symbols) and animals that grew slower than average were designated growth faltering (*n* = 10, red symbols). **b** Growth faltering animals had lower levels of serum tryptophan [27.0 ± 14.2 μM vs. 39.9 ± 11.9 μM (Mean ± SD)] and higher levels of kynurenine (3.78 ± 1.51 μM vs. 3.05 ± 1.20 μM) that together resulted in significant differences in K/T ratios (Kynurenine/Tryptophan ratio × 1000) in comparison with Healthy animals. **c** Shotgun metagenomic analysis of the luminal contents of the colon was used to compare the expression of microbial butyrate pathway genes (Mean ± SD of copies per million reads, healthy (*n* = 7, blue symbols), growth faltering (*n* = 9, red symbols). *P* values are based on unpaired student’s *t*-test. Source data are provided as a Source Data file.

To further characterize EED from a histological perspective, animals with a range of clinical histories were brought to necropsy for analysis of the entire GI tract. These groups represented the spectrum of infant enteric disease (or lack thereof) at each study site and included healthy animals with no history of diarrheal disease (*n* = 8), healthy animals that had recovered from a previous acute episode of diarrheal disease (*n* = 4, necropsied at 28–103 days post-recovery), and animals undergoing chronic, relapsing diarrheal disease (*n* = 5). Animals were stratified by their growth rate kinetics (Fig. 4a) according to a published standard curve^17^, with seven animals that grew faster than average (Healthy) and ten animals that grew slower than average (Growth Faltering). There were no clear differences in overall enteric pathogen exposure history between the Healthy and Growth Faltering groups (Supplementary Tables 3, 4). In addition to the xTAG analysis performed at 1 month (Fig. 1 and Supplementary Table 3), animals were screened for *Shigella* and *Campylobacter* by the microbial culture at 1, 3, 6, and 8 months of age as well as at necropsy since these represent two of the most common enteric pathogens encountered at each primate center (Supplementary Table 4). Shigella was rare, with no cases identified by microbial culture among the Healthy group at any time point and only two cases observed at single time points among the Growth Faltering group (one animal at 3 months and the other at 6 months of age, with negative cultures observed at subsequent time points). Based on sample availability, *Campylobacter* colonization was similar between the two groups with 43% (3/7) and 38% (3/8) *Campylobacter*-positive cultures observed at 1 month of age between the Healthy and Growth faltering animals, respectively whereas 86% (6/7) and 89% (8/9) of the animals in each group were *Campylobacter*-positive at necropsy. The *Shigella* and *Campylobacter* titers were not quantitatively determined by microbial culture but a recent microbiome study performed assembly of microbial genomes from the colonic luminal contents of the Healthy and Growth Faltering infants and this revealed a significant increase in the abundance of *Campylobacter* spp. genomes among Growth Faltering animals compared to Healthy controls^39^.

Differences in serum tryptophan and kynurenine levels were observed that together resulted in approximately twofold higher kynurenine/tryptophan (K/T) ratios among Growth Faltering animals compared to their Healthy counterparts (169 ± 91 vs. 82 ± 37, *P* = 0.03, Fig. 4b). These results are similar to the higher K/T ratios that have been associated with growth stunting among children in Tanzania^3^, Bangladesh^40,41^, and Brazil^42^. Although fecal butyrate levels were not measured in these studies, shotgun metagenomic analysis revealed significantly lower expression of colonic microbial butyrate pathway genes among Growth Faltering animals compared to Healthy controls (529 ± 242 vs. 842 ± 132, respectively; *P* = 0.009, Fig. 4c). These differences remained significant even when animals with active, chronic diarrhea are excluded from the Growth Faltering group and compared to Healthy controls (507 ± 306 vs. 842 ± 132, respectively; *P* = 0.026), indicating that colonic energy production may be altered in Growth Faltering infants regardless of current diarrheal status.

**Fig. 5:**
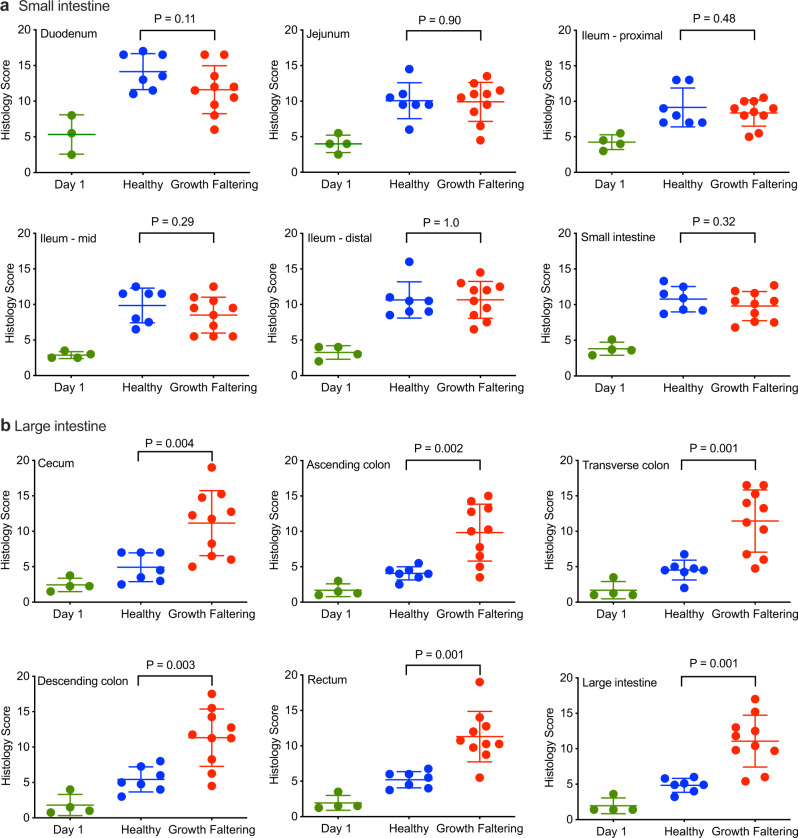
Histological evaluation of small and large intestine from healthy and growth faltering infant macaques. Histology scores were averaged from two blinded pathologists for each individual anatomical location in the small intestine as well as for the average of all sites within the small intestine (**a**) and at each individual location in the large intestine as well as for the average of all sites within the large intestine (**b**). Histology scores from infants at 1 day of age (Mean ± SD) are provided for comparison to the histology scores of older infants at 6–11 months of age (Mean ± SD) that were stratified into healthy and growth faltering groups based on their overall growth trajectories as outlined in Fig. 4. *P* values are based on a two-sided student’s *t*-test. Source data are provided as a Source Data file.

We next compared the blinded histological scores from the small intestine of Healthy vs. Growth Faltering animals (Fig. 5a and Table 3). Surprisingly, we found no significant differences between these two groups of animals in terms of histological abnormalities in the duodenum, jejunum, ileum (including proximal, middle, or distal ileum) or when the average histological values of the entire small intestine of each animal were calculated (*P* ≥ 0.11). In contrast to the small intestine, we found a greatly increased frequency of histological abnormalities in the large intestine of Growth Faltering infant macaques compared to their Healthy counterparts, including significant differences in the cecum, ascending colon, transverse colon, descending colon, rectum, and when the average histological values of the entire large intestine of each animal were calculated (*P* ≤ 0.004, Fig. 5b and Table 3). To complete the analysis of the entire GI tract, we also examined the esophagus (Supplementary Fig. 2a) and stomach (Supplementary Fig. 2b), but found no significant differences in histopathology between Healthy and Growth Faltering infant macaques at these anatomical sites (*P* = 0.82 and *P* = 0.61, respectively). A recent study found significantly increased expression of SMAD7 (a regulatory molecule in the TGF-β pathway associated with increased inflammation) in duodenal biopsies of Pakistani children with EED compared to healthy controls and celiac disease patients from Italy^43^. However, when we measured SMAD7 expression in the duodenum, jejunum, proximal ileum, transverse colon, and descending colon, we found no significant increase in expression among Growth Faltering infants compared to the Healthy controls who were living under identical exposure conditions (Supplementary Fig. 3). Since EED is a disease that occurs in the absence of clinically apparent diarrhea, it was possible that the inclusion of overtly diarrheal animals in the Growth Faltering group might have skewed the interpretation of the results as they specifically relate to EED. However, when animals with active, chronic diarrhea were removed from the analysis, there was still no significant difference in the histological findings observed among the various sites along the small intestine (*P* ≥ 0.28, Table 3), whereas histopathology scores still remained significantly higher among Growth Faltering animals in the absence of chronic diarrhea compared to Healthy animals at multiple anatomical locations including the cecum, ascending colon, transverse colon, rectum, and when the entire large intestine was analyzed (*P* ≤ 0.03, Table 3).

**Table 3:** Association between reduced growth kinetics and histological abnormalities in the small and large intestine.

GI Location		*P* value	*P* value (minus Diarrhea)
Small intestine	Duodenum	0.11	0.28
	Jejunum	0.9	0.42
	Ileum-proximal	0.48	0.71
	Ileum-mid	0.29	0.54
	Ileum-distal	1	0.97
	Small intestine	0.76	0.68
Large intestine	Cecum	0.004	0.02
	Ascending colon	0.002	0.02
	Transverse colon	0.001	0.03
	Descending colon	0.003	0.07
	Rectum	<0.001	0.004
	Large intestine	<0.001	0.01

At each of the indicated locations along the GI tract, *P* values were determined for histology scores observed between healthy (*n* = 7) and growth faltering animals (*n* = 10) or between healthy animals and growth faltering animals after excluding those with chronic diarrhea (*P* value minus diarrhea, *n* = 5). The results provided for the entire small intestine in each group was based on the average scores for each animal’s duodenum, jejunum, ileum-proximal, ileum-mid, and ileum-distal locations whereas the values provided for the large intestine were based on the average scores of each animal’s cecum, ascending colon, transverse colon, descending colon, and rectum. *P* values are based on a two-sided student’s *t*-test.

**Fig. 6:**
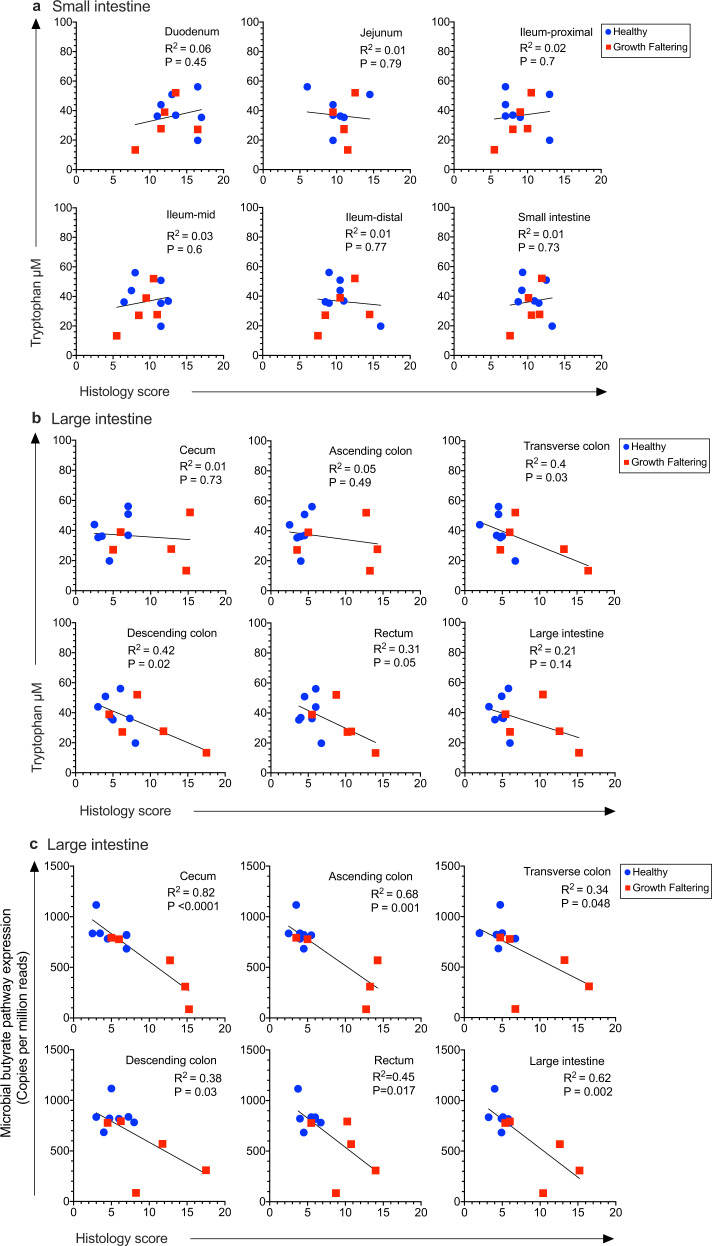
Association between serum tryptophan levels and microbial butyrate pathway gene expression with GI tract histopathology. The levels of serum tryptophan were compared to the degree of histological abnormalities for each individual anatomical location in the small intestine as well as for the average of all sites within the small intestine (**a**) and at each individual location in the large intestine as well as for the average of all sites within the large intestine (**b**) among healthy (*n* = 7, blue symbols) and growth faltering (*n* = 5, red symbols) infant macaques. **c** Microbial butyrate pathway gene expression was measured in comparison with histopathology of the large intestine among healthy (*n* = 7, blue symbols) and growth faltering (*n* = 5, red symbols) infant macaques. Animals with chronic diarrhea at the time of necropsy were excluded from analysis in order to focus on EED among clinically asymptomatic infant macaques. *P* values were determined by univariable linear regression. Source data are provided as a Source Data file.

To determine if the anatomical site of gut pathology was associated with nutritional uptake and systemic inflammation among infant macaques with EED, we plotted histopathology scores from the small and large intestine in comparison to circulating levels of tryptophan and kynurenine as well as the K/T ratios. Animals with active, chronic diarrhea at the time of necropsy showed evidence of systemic inflammation with significantly increased levels of circulating sCD14 compared to other Growth Faltering and Healthy animals^39^ and were excluded from this analysis in order to focus on infants with a non-diarrheal EED phenotype. There was no significant relationship between tryptophan levels and histological abnormalities at any location within the small intestine (*P* ≥ 0.45, Fig. 6a). In contrast, serum tryptophan levels were inversely correlated with histopathology in the transverse colon (*P* = 0.03), descending colon (*P* = 0.02), and rectum (*P* = 0.05, Fig. 6b). Inclusion of animals with active chronic diarrhea did not alter the results observed in the small intestine whereas, in the large intestine, the inverse correlation between serum tryptophan levels and histopathology in the transverse colon and the descending colon remained significant (*P* = 0.02 and *P* = 0.01, respectively; Supplementary Fig. 4). Serum kynurenine levels remained stable among the infant macaques regardless of the degree of gut pathology and were not associated with histological aberrations within the small intestine (*P* ≥ 0.36, Supplementary Fig. 5a) or large intestine (P ≥ 0.52, Supplementary Fig. 5b) or when chronic diarrheal animals were included in the analysis (*P* ≥ 0.18, Supplementary Fig. 6). Serum K/T ratios showed no association with histopathology of the small intestine (*P* ≥ 0.26, Supplementary Fig. 7a) but instead were significantly increased in association with higher histopathology scores in the transverse colon (*P* = 0.001), descending colon (*P* = 0.001), rectum (*P* = 0.01), and the complete large intestine (*P* = 0.01, Supplementary Fig. 7b). Inclusion of animals with chronic diarrhea did not change the K/T ratio vs. histopathology results observed in the small intestine whereas, in the large intestine, only the association of high K/T ratios and higher histopathology scores in the transverse colon and descending colon remained significant (*P* = 0.02 and *P* = 0.008, respectively, Supplementary Fig. 8).

Since Growth Faltering infants had significantly lower expression of microbial genes involved with butyrate production (Fig. 4c), we determined if this might also be associated with gut pathology by comparing butyrate pathway gene expression levels to histological abnormalities in the GI tract (Fig. 6c). We found significant correlations between colonic microbial butyrate pathway gene expression levels and histopathology at all sites measured in the large intestine including the cecum (*P* < 0.0001), ascending colon (*P* = 0.001), transverse colon (*P* = 0.048), descending colon (*P* = 0.03), rectum (*P* = 0.017), and the complete large intestine (*P* = 0.002), whereas there was no association between colonic microbial butyrate pathway expression levels and histopathology at any site measured in the small intestine (*P* ≥ 0.11, Supplementary Fig. 9) with similar patterns observed when animals with chronic diarrhea were included in the analysis (Supplementary Fig. 10). Together, these results indicate that there may be substantial large intestine involvement in EED since higher histopathology scores in the large intestine correlated significantly with lower serum tryptophan levels, higher K/T ratios, lower microbial expression of butyrate pathway genes, and poor infant growth trajectories.

## Discussion

In these studies, we determined the histopathology of the entire GI tract of non-human primates that were exposed to enteric pathogens at an early age and acquired an EED phenotype during infancy. This model not only allows determination of the extent of intestinal damage across the various compartments of the GI tract, but also allows one to determine the degree of histological aberrations and potential association with infant growth stunting. EED has been previously defined as a disease of the small intestine and we anticipated finding an association between poor growth trajectories and histopathology in the small intestine. However, histological abnormalities in the small intestine showed no correlation with infant growth characteristics and instead, significantly increased histopathology of the large intestine was associated with reduced growth trajectories, reduced levels of circulating tryptophan, and increased K/T ratios. Based on these unexpected findings, the importance of the large intestine in EED may represent a previously overlooked component of this complex enteric disease.

EED is not the only enteric disease that may be associated with villous atrophy. Because enterocytes in the small intestine have a short life span and are replaced every 3 to 4 days, villus atrophy is a common response of the small intestine to injury in a wide variety of situations where the balance between enterocyte loss from villi and replacement by stem cell division from the villus crypts is disrupted. Chronic enterocolitis and gluten sensitivity/celiac disease may also be associated with villous atrophy and although EED remains a poorly understood GI tract disease, it is distinct from chronic enterocolitis and celiac disease because EED is associated with villous atrophy that occurs in the absence of active diarrhea (Fig. 2b, c). Prior studies have shown that celiac disease is rare among rhesus macaques, with an incidence rate of ~1.2%^44^. Based on the observation that 100% (8/8) of 8–11-month-old infants showed evidence of villous atrophy in the absence of diarrhea or abdominal bloating (another sign of celiac disease among some juvenile macaques^36^), this indicates that the observed histological abnormalities in the small intestine (Fig. 2) were unlikely to be due to chronic enterocolitis or celiac disease. It would be interesting to quantitatively measure intake vs. absorbed protein, carbohydrate, and lipid-based calories among Healthy and Growth Faltering animals with differences in small and large intestine histopathology to determine the impact on subsequent nutrient absorption similar to studies performed with pediatric short bowel syndrome (SBS) patients^45^. Intestinal permeability is another important parameter associated with malnutrition that can be measured by the lactulose:mannitol (LM) test and this approach could provide further characterization of gut health among rhesus macaques with low or high histopathology in the small and large intestine, respectively.

Group-housed rhesus macaques provide an important model for the study of naturally acquired human enteric disease^16–19^. These animals have gut microbiomes^21–23^ that are similar to those observed among people living in resource-poor settings under conditions of poor sanitation and hygiene and are endemically exposed to enteric pathogens associated with human growth stunting, including *Campylobacter*, *Shigella*, ETEC, *Cryptosporidium*, *E. histolytica*, and *Giardia* (Fig. 1). *Campylobacter* infection is common among infant macaques (Fig. 1) and both symptomatic and asymptomatic carriage of *Campylobacter* has been associated with growth stunting of human infants^8^. The luminal contents of the colon from Growth Faltering infants also showed a significantly higher abundance of *Campylobacter* spp. genomes compared to Healthy controls^39^. Together, these results suggest a potential cause-and-effect relationship in which *Campylobacter* is an important environmental pathogen that is associated with infant growth stunting.

One of the major knowledge gaps in EED is a comprehensive analysis of associated gut pathology along the entire GI tract. Here, we have leveraged this unique animal model to shed light on this important clinical condition by providing a window into the underlying causes of EED. As expected, we identified villous blunting, atrophy, and reduced crypt-to-villus ratios in the small intestine similar to that observed among stunted children with EED^35,37,38^ (Fig. 2). However, these observations did not coincide specifically with poor growth kinetics (Fig. 5a). The lack of significant differences in small intestine pathology appears to be further supported by recent clinical studies involving histological assessment of the small intestine of growth-stunted children that also did not identify a significant association between duodenal biopsy scores and anthropometric parameters including height-for-age *Z*-scores or weight-for-height standard deviation *Z*-scores^35^. In other words, children with varying degrees of growth stunting could not be distinguished from each other based on histological analysis of the small intestine. The original postmortem histology study by Chacko et al., also noted that “Two children in our study suffered from protein-calorie malnutrition, but their mucosal architecture was no different from that of the other children”^37^. One provocative interpretation of these clinical studies is that the extent of villous atrophy observed in the small intestine may not be directly proportional to the degree of growth stunting. Histological abnormalities in the small intestine may be common among children living under conditions of poor sanitation and hygiene, but it is possible that this is not the sole driving factor involved with the complications of EED or severe malnutrition. In contrast to the observation of histopathology in the small intestine noted among all of the infant macaques in our study (Figs. 2,  5a), an EED phenotype of poor growth trajectory was only observed among infants with significantly increased histopathology in the large intestine (Fig. 5b and Table 3). This raises many intriguing questions regarding the potential role of the large intestine for digestive health under conditions of reduced nutritional uptake by the small intestine.

Among healthy individuals, the colon is not considered a major source of nutritional energy and is presumed to mainly function by absorbing water, vitamins, and electrolytes from undigested waste material that has passed through the small intestine. Colonic energy production occurs through fermentation of resistant starch and non-starch polysaccharides to short-chain fatty acids (SCFA) but this is believed to account for only 5–10% of energy absorption^45,46^. However, these calculations are based on a protein-rich European diet^47^ whereas dietary fiber intake may be up to sevenfold higher in developing countries^48^ and concomitantly results in a much higher proportion of total energy absorption through colonic fermentation compared to a low-fiber/high-protein diet^49,50^. Indeed, for other omnivore species like pigs that have GI tract morphology similar to humans^49^, the dietary energy obtained via the production of SCFA in the large intestine may be as high as 25% of total energy absorption^49^. Butyrate is one of the major SCFA produced in the large intestine and is not only the primary energy source for intestinal epithelia^51^ but is needed to maintain healthy epithelial integrity, villous architecture, and surface area^52,53^, which are all important for efficient nutrient absorption. Since microbial fermentation in the large intestine is the main source of this SCFA, disruption or loss of butyrate production/absorption could further exacerbate small intestine disease and malnutrition. For example, among children who died of severe acute malnutrition (SAM), butyrate concentrations were only 1.5% of normal levels (31 vs. 2,036 ng/mL, *P* = 0.02)^54^. Although we did not measure butyrate levels at the time of the study, the significant reduction in microbial expression of butyrate pathway genes among Growth Faltering infant macaques (Fig. 4c) and the significant inverse association between butyrate pathway gene expression and histopathology in the large intestine (Fig. 6c) appear to indicate a role for butyrate as an important energy source that may be lacking among growth-stunted children with EED. Since EED is most commonly observed among populations subsisting on low-protein/high-fiber diets in developing countries, the impact on dysregulated SCFA-based energy metabolism in the large intestine could be more important than previously realized.

Although a healthy colon absorbs roughly 140–180 kilocalories per day on a European-style diet^49^, it can absorb up to 1000 kilocalories per day in patients with malabsorption^55^. Compelling evidence for the importance of the colon/large intestine in energy absorption/salvage under conditions of malabsorption can be found among cases of pediatric SBS in which the absorptive surface of the small intestine has been sharply reduced due to surgical resection^45^. Comparison of patients with short but similar small intestine lengths have shown that those with a functioning colon have improved nutritional energy preservation compared to those who do not have a colon^46,56^. Moreover, other studies indicate that approximately half of the energy needs may be provided by the colon^46^ and SBS patients who do not have a colon have a poor prognosis^45^. Following massive resection of the small intestine, an adaptive process will take place and over time manifests in reduced diarrheal episodes and increased nutritional uptake by the large intestine. One adaptation is that the human colorectal mucosa will express glucose transporters (e.g., GLUT2, SGLT1, and GLUT5)^57^, representing a potential mechanism for monosaccharide uptake under conditions of high carbohydrate exposure in the large intestine when there is poor nutrient absorption by the small intestine. Likewise, the peptide transporter, PepT1, is upregulated by as much as fivefold in the colonic mucosa of SBS patients, indicating a mechanism for enhanced luminal di- and tripeptide transport^58^. PepT1 protein expression was found only on absorptive colonocytes (and not by goblet cells) along the colonic crypt^58^ and following transport into cells, these small peptides undergo intracellular hydrolysis to free amino acids for delivery into portal blood^59^. Together, these studies indicate that under certain disease conditions, the colon may play a more important role in nutrient uptake as well as SCFA production, both of which may be critical in cases of EED wherein nutrient absorption in the small intestine may be compromised.

Among human infants, serum tryptophan concentrations are directly associated with linear growth from 1 to 8 months after analysis^3^. Indeed, every 1 standard deviation increase in tryptophan level was associated with a 0.10 (Peru) to 0.13 (Tanzania) gain in length-for-age Z (LAZ) score of infants over the next 6 months. This indicates that tryptophan is an important and easily measured biomarker for potential future growth. The relationship between tryptophan and protein synthesis has been studied extensively in pigs since it is known to be a growth-limiting amino acid, especially for animals on corn-based feed^60^ and this could have important implications for humans in resource-poor settings who live primarily on corn-based diets. Even moderate tryptophan deficiency will cause a reduction in porcine growth rates that is compounded by a reduction in appetite/feed intake, an outcome not commonly observed for other limiting amino acids except for valine^60^. In our studies, we found that Growth Faltering infants had 32% lower circulating levels of tryptophan on average compared to Healthy infants along with modestly higher kynurenine levels that together resulted in a significant difference in K/T ratios (Fig. 4). High K/T ratios have been described in other inflammatory diseases and tryptophan degradation to kynurenine due to inflammation-induced upregulation of indoleamine 2,3-dioxygenase 1 (IDO1) has been implicated in this process^3^. It is also possible that lower tryptophan levels in circulation could be at least partly due to poor nutrient absorption in the large intestine, especially among individuals with increased histopathology at these sites (Fig. 6b). Moreover, loss of butyrate production/absorption in the large intestine could further exacerbate small intestine disease by depriving intestinal epithelial cells of an important energy source.

Under normal digestive conditions, most nutrient absorption occurs in the small intestine but if the small intestine is compromised and excess nutrients pass through to the colon, then the large intestine may act as an energy salvage organ. In the case of EED, our hypothesis is that when this secondary energy salvage mechanism is also impaired (i.e., demonstrated by increased histopathology), then the risk of infant growth stunting will increase in parallel. More work is clearly needed to understand the underlying GI tract pathology that may be contributing to poor nutritional uptake and subsequent infant growth stunting but the results presented here indicate that both the small and large intestine may be important in this process.

## Methods

### Rhesus macaques

This study was performed in strict accordance with the recommendations described in the Guide for the Care and Use of Laboratory Animals of the National Institute of Health, the Office of Animal Welfare, and the United States Department of Agriculture. All animal work was approved by the Oregon National Primate Research Center (ONPRC) Institutional Animal Care and Use Committee (IACUC protocol IP00000416) and the California National Primate Research Center (CNPRC) Institutional Animal Care and Use Committee (IACUC protocol 19234). Both Centers are accredited by the Association for the Assessment and Accreditation of Laboratory Animal Care International. Rhesus macaques (*Macacca mulatta*) were housed outdoors in groups and fed twice daily with a standard commercial primate chow with water available ad libitum. Forty infants from each primate center (*n* = 80 infants, total) were weighed, measured, and screened for enteric pathogens at 1 month of age. Next, 20 infants from each primate center (the top 25th percentile and bottom 25th percentile based on weight) were monitored longitudinally for growth rate kinetics and diarrheal episodes with rectal swabs screened for *Campylobacter* and *Shigella* by the microbial culture at 1, 3, 6, and 8 months of age. Based on growth rates and incidence of acute and chronic diarrhea, a panel of animals was selected for necropsy and comprehensive histological analysis (Supplementary Fig. 1). At 8–11 months of age, eight infants (four from each primate center), representing the top 25th percentile and bottom 25th percentile of longitudinally monitored infants based on weight and no history of diarrhea were selected for histological analysis. In addition, four infants that had recovered from a single episode of acute diarrhea in the distant past (two infants from each primate center, examined at 28, 41, 77, and 103 days after recovery) and five infants (two from CNPRC and three from ONPRC) with active, chronic diarrhea at the time of necropsy were selected for histological evaluation. All procedures, including blood draws and rectal swabs were performed under ketamine or Telazol anesthesia by trained personnel under the supervision of veterinary staff. For necropsy procedures, animals were humanely euthanized by veterinary staff in accordance with regulatory guidelines. Euthanasia was conducted under anesthesia with ketamine followed by an overdose with sodium pentobarbital. This method is consistent with the recommendation of the American Veterinary Medical Association.

### xTAG and microbial cultures

Rhesus macaques had rectal swabs taken at each visit and were evaluated by the direct microbial culture at each respective site’s clinical pathology laboratory as well as by xTAG GPP (Luminex) analysis by PCR following the manufacturer’s instructions. Only samples that scored positive in two independent xTAG experiments were considered positive. For microbial cultures, the ONPRC clinical pathology laboratory routinely screened for *C. coli*, *C. jejuni*, *S. flexneri*, *S. dysenteriae*, and *Yersinia*. The CNPRC clinical laboratory screened for *C. coli*, *C. jejuni*, *C. lari*, *S. flexneri*, *S. dysenteriae*, and *Yersinia*. CNPRC also tested for *Salmonella* during diarrheal clinical visits. Direct microbial culture-based detection of *Campylobacter* was more sensitive than the xTAG-based approach and therefore only the microbial culture-based results for *Campylobacter* are provided.

### Histology

Tissue samples for each animal were collected from the esophagus, stomach, duodenum, jejunum, proximal ileum, mid ileum, distal ileum, cecum, ascending colon, transverse colon, descending colon, and rectum. The tissues were placed in 10% neutral buffered formalin at room temperature for 48–72 h and processed in a Tissue Tek VIP 5 Tissue Processor. The blocks were paraffin-embedded, cut, and stained with hematoxylin and eosin. The slides were randomized, blinded, and shipped to two pathologists for independent evaluation based upon pre-determined scoring criteria (Tables 1,  2 and Supplementary Tables 1,  2). Histology scores calculated from the published work by Chacko et al.^37^ were determined by placing a grid overlay on their data in order to estimate individual numbers for fetuses and infants/small children using their histology grading system (1; fingers, 2; tongues; 3; leaves, 4; ridges, and 5; convolutions).

### Serum tryptophan and kynurenine measurements

Serum tryptophan and kynurenine levels were measured by the ONPRC Endocrine Technologies Support Core (ETSC) using ultra-high-performance liquid chromatography-heated electrospray ionization-tandem triple quadrupole mass spectrometry (LC-MS/MS) on a Shimadzu Nexera-LC-MS-8050 instrument (Kyoto, Japan). For sample preparation, 10 μl of the sample was combined with 10 μl of an internal standard mixture (10 μg/ml tryptophan-d3 in 0.1% formic acid) and extracted with 100 μl of cold methanol for 30 min on ice. Following extraction, samples were centrifuged, filtered, dried under forced air, reconstituted in 100 ul of 5:95 methanol:water with 0.1% formic acid, and placed onto 96-well microtiter plates. A quality control pool of rhesus macaque serum was prepared in the same manner as samples and assayed in quadruplicate on each plate. A six-point standard curve from 40 to 0.0128 μg/ml was prepared in charcoal-stripped human serum (Biochemed Services, Winchester, VA) demonstrated to be free of tryptophan and kynurenine. Standard curve points were extracted and assayed in triplicate in the same manner as the samples. A quality control sample containing 25 ng/ml of each standard in 5:95 methanol:water with 0.1% formic acid was run daily before and after each assay to confirm system suitability.

After the reconstitution step, samples were subjected to LC-MS/MS analysis. Using a Shimadzu SIL-30CAMP autosampler set to 10 °C, 5 μl of the sample was injected onto an Ace Excel 2 C18-PFP column (50 mm × 2.1 mm). Chromatographic separation occurred at 15 °C at a flow rate of 500 μl/min. Using a Shimadzu Nexera LC30-AD system, a linear gradient starting at 5% mobile phase B and ending at 95% mobile phase B was run for 2.25 min after a 0.30-min hold at 5% B. Mobile phase A (0.1% formic acid, pH 2.6) and mobile phase B (0.025% formic acid, 5 mM ammonium formulated in methanol) were chosen after optimization to minimize carryover while also maximizing detector signal and column retention. Heated electrospray ionization (ESI) interface settings were optimized for signal and stability. The interface temperature was 300 °C, the desolvation line temperature was 200 °C, and the heat block temperature was 500 °C. Gas was supplied by a Peak Genius 1051 nitrogen and air generator. Nitrogen gas was used for nebulizing and drying gases, while the air was used for heating gas. Nebulizing gas flow was set at 2 L/min, the heating gas flow was set at 10 L/min, and the drying gas flow was set at 10 L/min. Interface voltage was set to 1 kV. Scheduled multiple reaction monitoring (MRM) transitions were collected using a Shimadzu LC-MS-8050 tandem triple quadrupole MS in positive mode with two MS transitions for each analyte at their respective retention times: tryptophan (205.00 > 146.10, 205.00 > 118.20) at 1.98 min, kynurenine (209.10 > 94.15, 209.10 > 146.05) at 1.49 min, and tryptophan-d3 (208.15 > 147.00, 208.15 > 119.15) at 1.98 min. Data processing and analysis was performed using LabSolutions Software, V5.72 (Shimadzu, Kyoto, Japan). The extraction recovery was 80% for both amino acids. Intra-assay variation based on the rhesus macaque QC pool was less than 5% and inter-assay variation was less than 6% for both tryptophan and kynurenine (*n* = 4).

### SMAD7

Tissues were lysed in 1X Cell Lysis buffer (Cell Signaling Technologies) in a Qiagen Tissuelyser with stainless steel beads (McMaster) and then subjected to three freeze/thaw cycles, and centrifuged at 10,000 × *g* for 10 min at 4 °C. Supernatants were collected and protein concentration determined using the DC protein assay (Bio-Rad). Before loading onto 4–12% SDS-PAGE, dithiothreitol was added to equal amounts of sample for a final concentration of 100 mM and bromophenol blue to a final concentration of 0.1% and boiled. After running the SDS-PAGE, the protein was transferred to a nitrocellulose membrane. The membrane was blocked in 5% milk/TBS-T and incubated overnight in primary antibody (mouse monoclonal antibody, B-8, Santa Cruz Biotechnology) at 4 °C at 1:500 in 5% BSA/TBS-T. Secondary antibody incubation (mouse monoclonal antibody, 65 C, Santa Cruz Biotechnology) was performed at 1:5000 in 5% milk/TBS-T for 1 h. Signal was detected using SuperSignal West Femto substrate (ThermoFisher Scientific) and visualized on a Fluorchem HQ (Protein Simple). SMAD7 signal was normalized to GAPDH and subsequently normalized across gels to a control sample. Mouse monoclonal antibody SMAD7 (B-8) and mouse monoclonal GAPDH(6C5) were purchased from Santa Cruz Biotechnology.

### Shotgun metagenomics

Total DNA was extracted from ~0.25 gm of colonic luminal contents using the PowerSoil DNA Isolation Kit (MO BIO Laboratories, Carlsbad, CA, USA) as previously described^39^. Shotgun metagenomic libraries were prepared from 50 ng of genomic DNA using a Nextera library (Illumina, La Jolla CA) prepared per Illumina’s recommended protocol and sequenced on an Illumina HiSeq 4000 2 × 100 using HiSeq Control software HD3.4.0. Raw demultiplexed reads were quality filtered, and potential host reads were removed by aligning trimmed reads to the *Macaca mulatta* genome (Mmul 8.0.1). Trimmed and decontaminated reads generated from the transverse and descending colon of each animal were concatenated to increase sequencing depth. Reads were then functionally annotated using the HUMAnN2 pipeline using default settings with the UniRef50 database and assigned to Metacyc pathways. An abundance of microbial butyrate-related gene expression, also described as the “pyruvate fermentation to butanoate” pathway (genes: pfoA, thl, hbd, crt, etfA, etfB, bcd, ptb, buk1), was determined by the least abundant gene in the pathway (i.e., a minimum number of complete pathways) and normalized using copies per million (CPM) reads.

### Statistics

A two-sided student’s *t*-test was used to compare the mean histology scores, SMAD7 levels, and K/T ratios between Healthy and Growth Faltering infant groups. There was no adjustment for multiple comparisons. For determining potential associations between histology scores and tryptophan, kynurenine, or K/T ratios, coefficient of determination (*R*^2^) and *p* values from univariable linear regression were reported. For measures of agreement between histology scores from the two pathologists, we reported both coefficient of determination (*R*^2^) and concordance correlation coefficient (CCC).

### Reporting summary

Further information on research design is available in the Nature Research Reporting Summary linked to this article.

## Supplementary information

Supplementary Information

Reporting summary

## Data Availability

The data supporting the results of this study are available within the Source data files provided with this paper. Shotgun metagenomics study reads were functionally annotated using the HUMAnN2 pipeline using default settings with the UniRef50 database. The sequencing datasets used in this study are available in the NCBI SRA repository, under the bioproject ID no. PRJNA729051. Digitized images of the histology slides described in this study have been archived at the Washington University Digital Pathology Exchange (WUPAX). Source data are provided with this paper.

## Source data

Source Data
