# Supplementary material for: Reduced infant rhesus macaque growth rates due to environmental enteric dysfunction and association with histopathology in the large intestine
Source: Nat Commun. 2022 Jan 11;13:234. doi: 10.1038/s41467-021-27925-x (PMC8752659; doi:10.1038/s41467-021-27925-x)
Supplement: Supplementary file 1 — Supplementary Information [file 41467_2021_27925_MOESM1_ESM.pdf]

Supplementary Table 1. Criteria and assigned values for microscopic changes in the esophagus

Value	0	1	2	**3**
Bacteria	Absent	Present		
Inflammation	Normal	Mild	Severe	

Supplementary Table 2. Criteria and assigned values for microscopic changes in the stomach

Value	0	1	2	3
Fibrosis in lamina propria	Absent	Present		
Lymphocytic gastritis	Normal	Mild	Severe	
Eosinophils	Absent	Present		
Lympho-follicular hyperplasia	Absent	Minimal	Prominent	

Supplementary Table 3. Enteric pathogens at 1 month of age

Animal #	Group	Site	Campylobacter Culture	ETEC xTAG	Shigella xTAG	Cryptosporidium xTAG	Entamoeba histolytica xTAG	Giardia xTAG
1	Healthy	ONPRC	Pos	Neg	Neg	Neg	Neg	Pos
2	Healthy	ONPRC	Pos	Neg	Pos	Neg	Neg	Pos
3	Healthy	ONPRC	Pos	Neg	Neg	Neg	Neg	Neg
4	Healthy	CNPRC	Neg	Neg	Neg	Neg	Neg	Pos
5	Healthy	CNPRC	Neg	Neg	Neg	Neg	Neg	Pos
6	Healthy	CNPRC	Neg	Pos	Neg	Neg	Neg	Neg
7	Healthy	CNPRC	Neg	Neg	Neg	Neg	Pos	Pos
8	Faltering	ONPRC	Pos	Neg	Neg	Neg	Neg	Neg
9	Faltering	ONPRC	Pos	Neg	Neg	Neg	Neg	Neg
10	Faltering	CNPRC	Neg	Neg	Neg	Neg	Neg	Neg
11	Faltering	CNPRC	Neg	Neg	Pos	Neg	Pos	Pos
12	Faltering	CNPRC	Neg	Neg	Neg	Neg	Neg	Pos
13	Faltering/CD	ONPRC	Pos	Neg	Neg	Neg	Neg	Neg
14	Faltering/CD	ONPRC	No data	No data	No data	No data	No data	No data
15	Faltering/CD	ONPRC	Neg	Neg	Neg	Neg	Neg	Neg
16	Faltering/CD	CNPRC	Neg	Neg	Pos	Neg	Pos	Neg
17	Faltering/CD	CNPRC	No data	No data	No data	No data	No data	No data

CD: Chronic Diarrhea

Supplementary Table 4. Longitudinal analysis of Campylobacter and Shigella by microbial culture

			Campylobacter Culture					Shigella Culture				
Animal #	Group	Site	1 month	3 month	6 month	8 month	Necropsy	1 month	3 month	6 month	8 month	Necropsy
1	Healthy	ONPRC	Pos	Pos	Pos	Pos	Pos	Neg	Neg	Neg	Neg	Neg
2	Healthy	ONPRC	Pos	Pos	Neg	Pos	Pos	Neg	Neg	Neg	Neg	Neg
3	Healthy	ONPRC	Pos	No data	No data	Pos	Pos	Neg	No data	No data	Neg	Neg
4	Healthy	CNPRC	Neg	Neg	Pos	Pos	Neg	Neg	Neg	Neg	Neg	Neg
5	Healthy	CNPRC	Neg	Pos	Pos	Pos	Pos	Neg	Neg	Neg	Neg	Neg
6	Healthy	CNPRC	Neg	Neg	Pos	Pos	Pos	Neg	Neg	Neg	Neg	Neg
7	Healthy	CNPRC	Neg	Pos	Pos	Pos	Pos	Neg	Neg	Neg	Neg	Neg
8	Faltering	ONPRC	Pos	Neg	Pos	Pos	Pos	Neg	Neg	Neg	Neg	Neg
9	Faltering	ONPRC	Pos	Neg	Pos	Pos	Pos	Neg	Neg	Neg	Neg	Neg
10	Faltering	CNPRC	Neg	No data	No data	Pos	Pos	Neg	No data	No data	Neg	Neg
11	Faltering	CNPRC	Neg	Pos	Pos	Pos	Pos	Neg	Neg	Neg	Neg	Neg
12	Faltering	CNPRC	Neg	Pos	Neg	Pos	Pos	Neg	Neg	Neg	Neg	Neg
13	Faltering/CD	ONPRC	Pos	No data	No data	No data	Pos	Neg	No data	No data	No data	No data
14	Faltering/CD	ONPRC	No data	No data	No data	No data	Neg	No data	No data	No data	No data	Neg
15	Faltering/CD	ONPRC	Neg	Pos	Pos	Neg	Pos	Neg	Pos	Neg	Neg	Neg
16	Faltering/CD	CNPRC	Neg	Neg	Neg	Neg	Neg	Neg	Neg	Pos	Neg	Neg
17	Faltering/CD	CNPRC	No data	No data	No data	No data	No data	No data	No data	No data	No data	No data

CD: Chronic Diarrhea

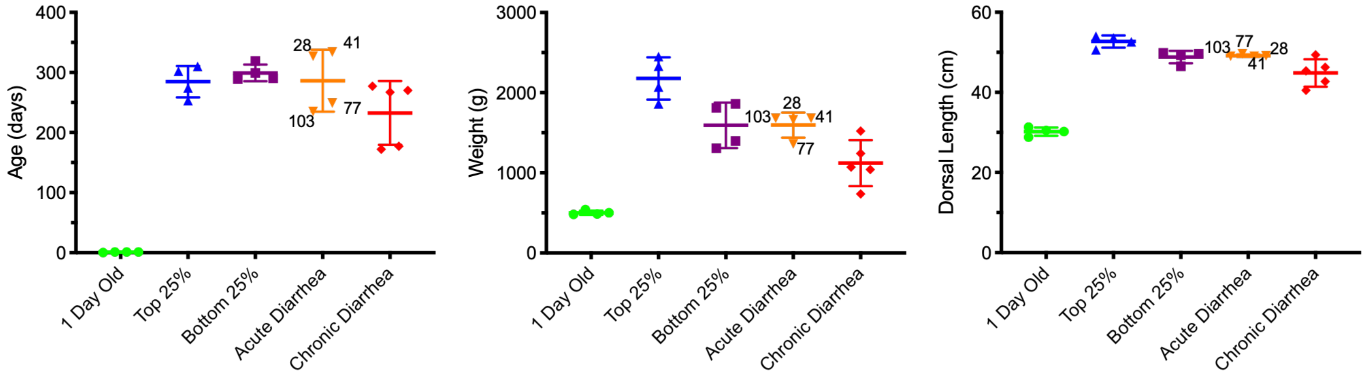

Supplementary Fig. 1. Selection of infants for determining EED characteristics based on GI tract pathology. Infant groups included infants at 1 day of age (1 Day Old, n=4, green symbols), large infants with no history of diarrhea selected from the top 25th percentile by weight at 8-10 months of age (Top 25%, n=4, blue symbols), small infants with no history of diarrhea selected from the bottom 25th percentile by weight at 8-10 months of age (Bottom 25%,n=4, purple symbols), infants with a history of acute diarrhea (Acute Diarrhea, n=4, yellow symbols), and infants with active, chronic diarrhea requiring humane euthanasia (Chronic Diarrhea, n=5, red symbols). The panels represent the age, weight, and dorsal length of the animals at the time of necropsy (Mean±SD). The numbers shown near the symbols of the Acute Diarrhea group represent the number of days between recovery from acute diarrhea and the date of necropsy. Source data are provided as a Source Data file.

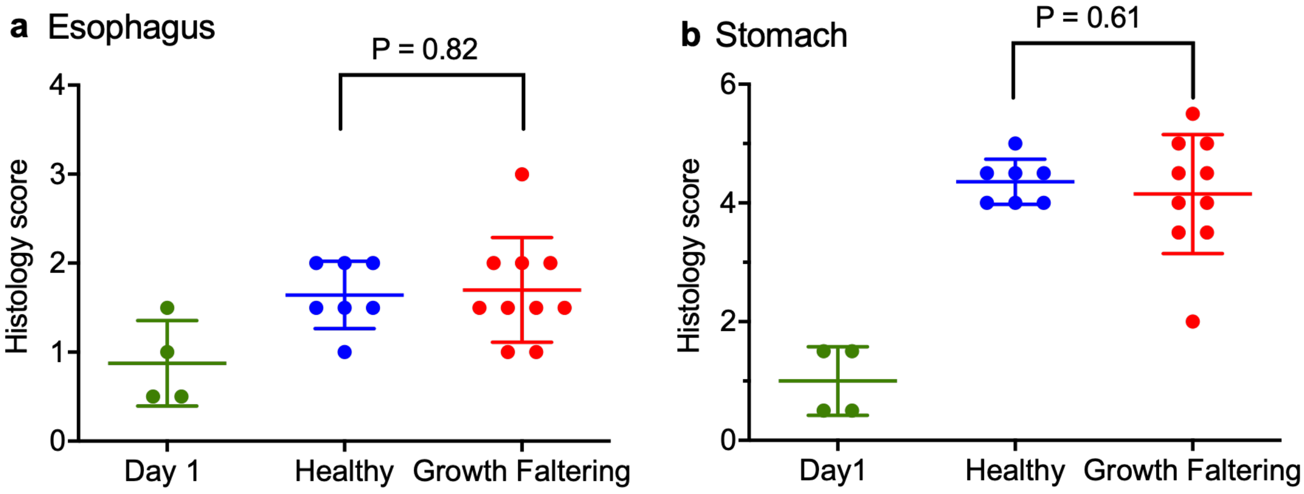

Supplementary Fig. 2. Blinded histological analysis of the esophagus and stomach. Quantitation of histology scores were averaged from two blinded pathologists for the esophagus (A) and stomach (B) using scoring criteria outlined in Supplementary Table 3 and Supplementary Table 4, respectively. Histology scores from infants at 1 day of age (Mean±SD, n=4, green symbols) are provided for comparison to the histology scores of older infants at 6-11 months of age (Mean±SD) that were stratified into Healthy and Growth Faltering groups based on their overall growth trajectories as outlined in Fig. 3. Although there was no statistically significant difference in esophagus histology scores between Healthy(n=7, blue symbols) and Growth Faltering(n=10, red symbols) infant macaques (P = 0.82), the esophagus histology scores of Healthy and Growth Faltering infants were significantly higher than that observed at 1 day of age (P = 0.016 and P = 0.029, respectively). Likewise, although there was no statistically significant difference in stomach histology scores between Healthy and Growth Faltering infant macaques (P = 0.61), the stomach histology scores of Healthy and Growth Faltering infants were significantly higher than that observed at 1 day of age (P < 0.0001 and P < 0.0001, respectively). P values were determined using two-sided student's t-test. Source data are provided as a Source Data file.

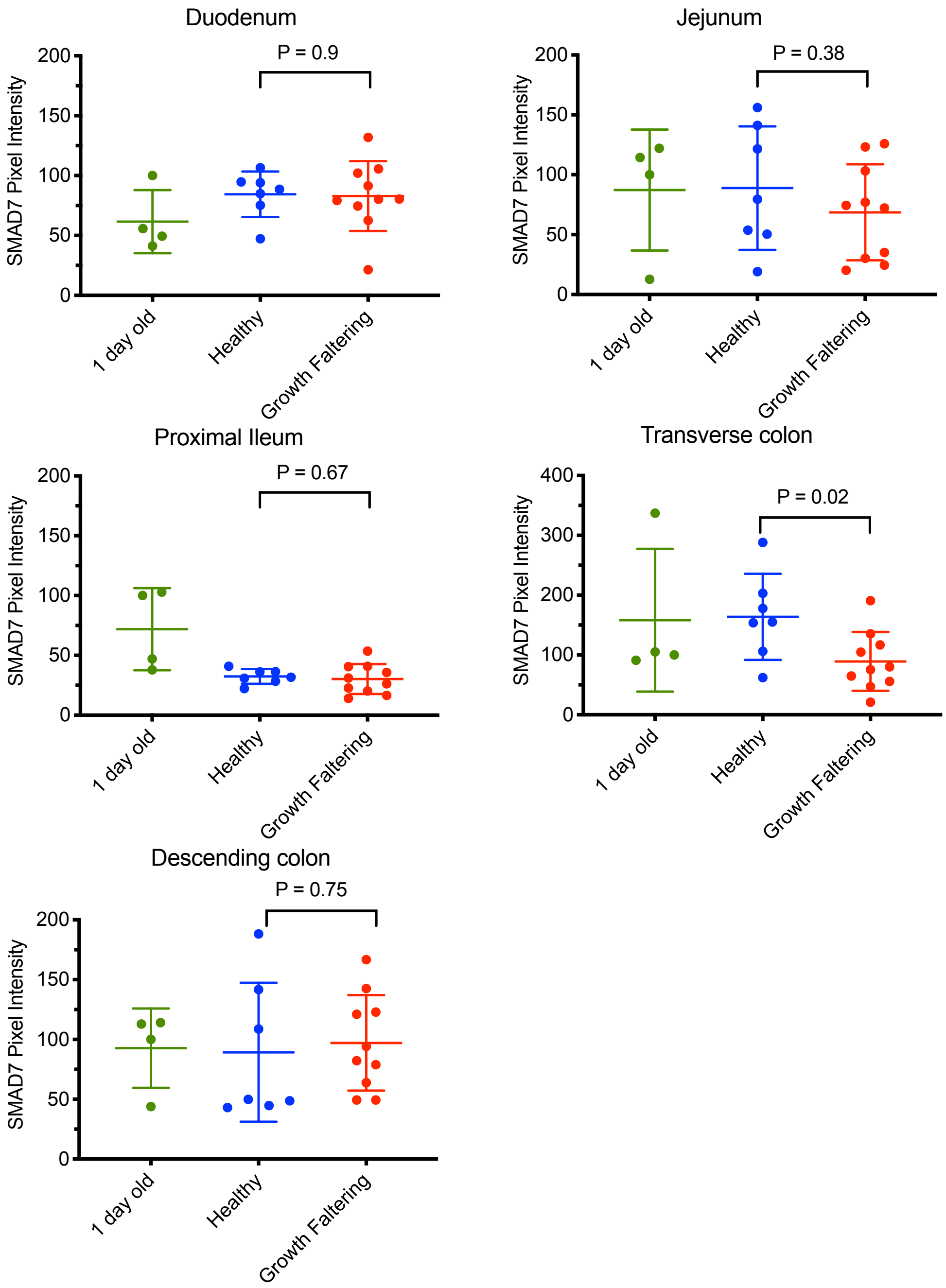

Supplementary Fig. 3. SMAD7 expression in the small and large intestine of Healthy and Growth Faltering infant macaques.

Tissue samples from each of the selected anatomical sites were lysed, loaded onto 4-12% SDS-PAGE and the concentration of SMAD7 was measured by Western blot after normalization to GAPDH as described in the Online Methods. SMAD7 expression is compared ((Mean±SD) between 1 day old (n=4, green symbols), Healthy (n=7, blue symbols) and Growth Faltering (n=10, red symbols) groups. P values were determined using two-sided student's t-test. Source data are provided as a Source Data file.

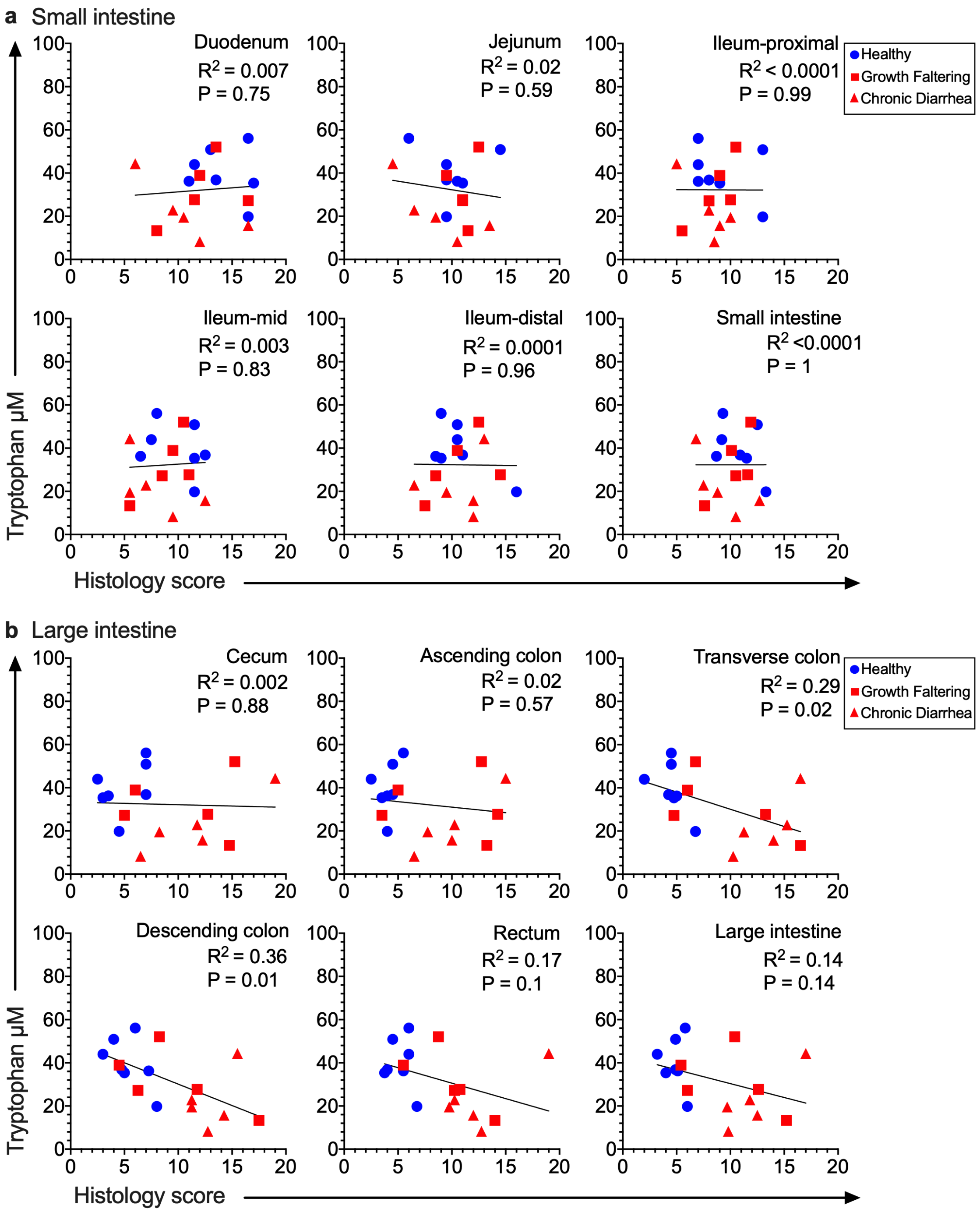

Supplementary Fig. 4. Inclusion of animals with chronic diarrhea (Healthy (n=7, blue circles), Growth Faltering (n=10, red symbols), Chronic diarrhea (n=5, red triangles)in measuring the association between histological scores and serum tryptophan levels. P values were determined by univariable linear regression. Source data are provided as a Source Data file.

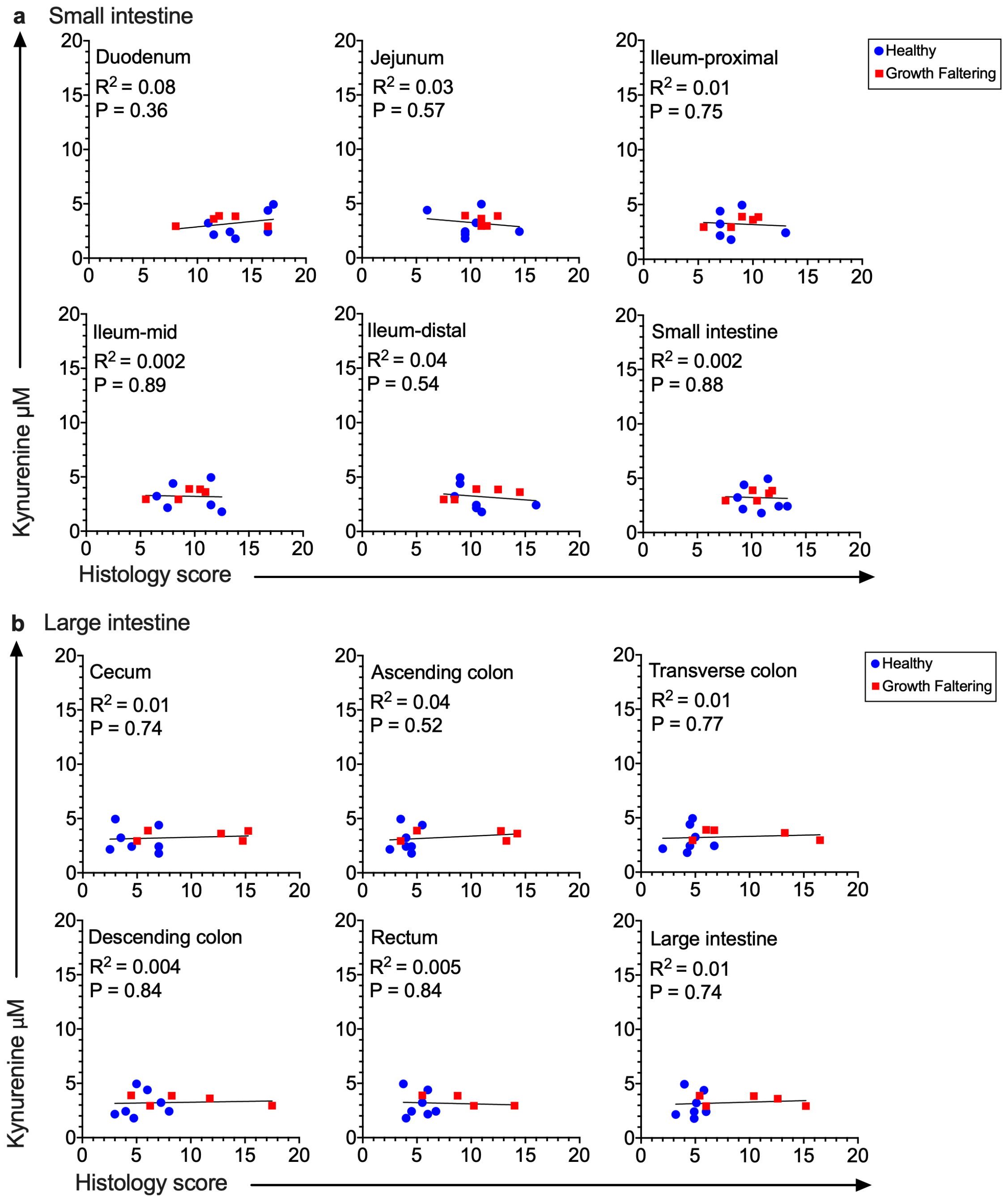

Supplementary Fig. 5. Association between histological scores and serum kynurenine levels. The levels of serum kynurenine were compared to the degree of histological abnormalities for each individual anatomical location in the small intestine as well as for the average of all sites within the small intestine (A) and at each individual location in the large intestine as well as for the average of all sites within the large intestine (B) among Healthy (n=7, blue symbols) and Growth Faltering(n=5, red symbols) infant macaques. Animals with chronic diarrhea at the time of necropsy were excluded from analysis in order to focus on EED and serum kynurenine levels among clinically asymptomatic infant macaques. P values were determined by univariable linear regression. Source data are provided as a Source Data file.

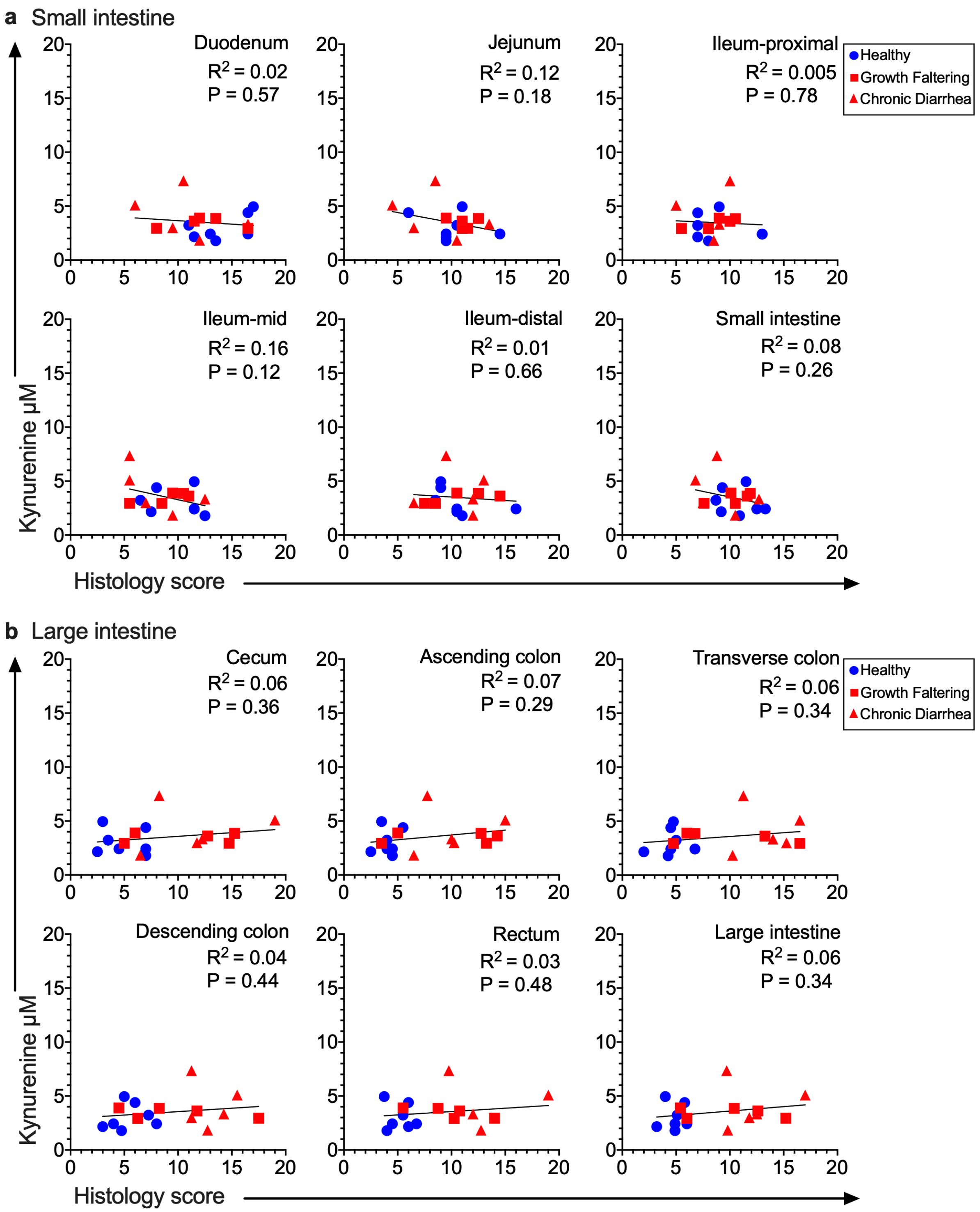

Supplementary Fig. 6. Inclusion of animals with chronic diarrhea in measuring the association between histological scores and serum kynurenine levels (Healthy (n=7, blue circles), Growth Faltering (n=10, red symbols), Chronic diarrhea (n=5, red triangles).P values were determined by univariable linear regression. Source data are provided as a Source Data file.

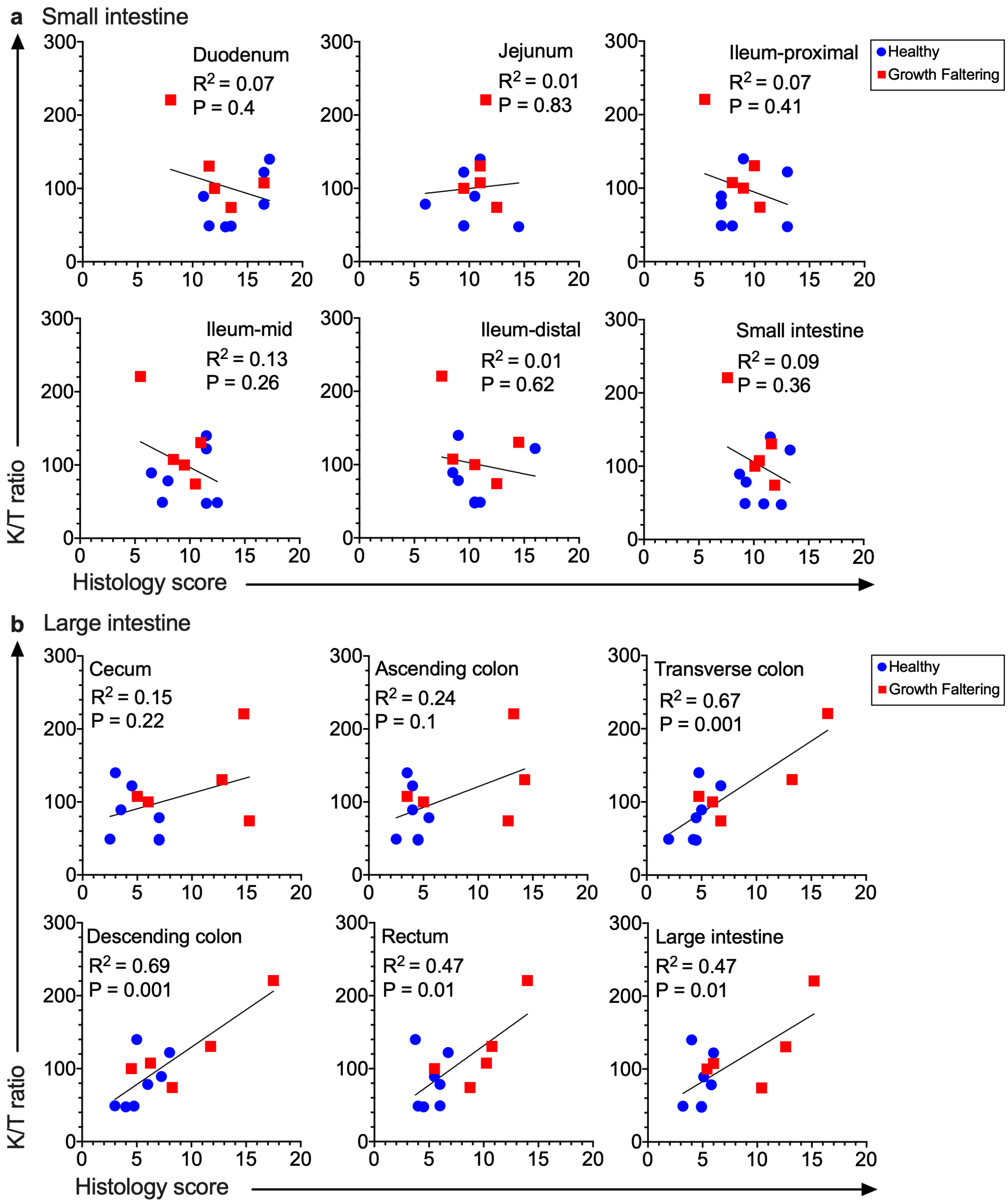

Supplementary Fig. 7. Association between histological scores and K/T ratios.

The K/T ratios (x 1,000) were compared to the degree of histological abnormalities for each individual anatomical location in the small intestine as well as for the average of all sites within the small intestine (A) and at each individual location in the large intestine as well as for the average of all sites within the large intestine (B) among Healthy(n=7, blue symbols) and Growth Faltering(n=5, red symbols) infant macaques. Animals with chronic diarrhea at the time of necropsy were excluded from analysis in order to focus on EED and K/T ratios among clinically asymptomatic infant macaques. P values were determined by univariable linear regression. Source data are provided as a Source Data file.

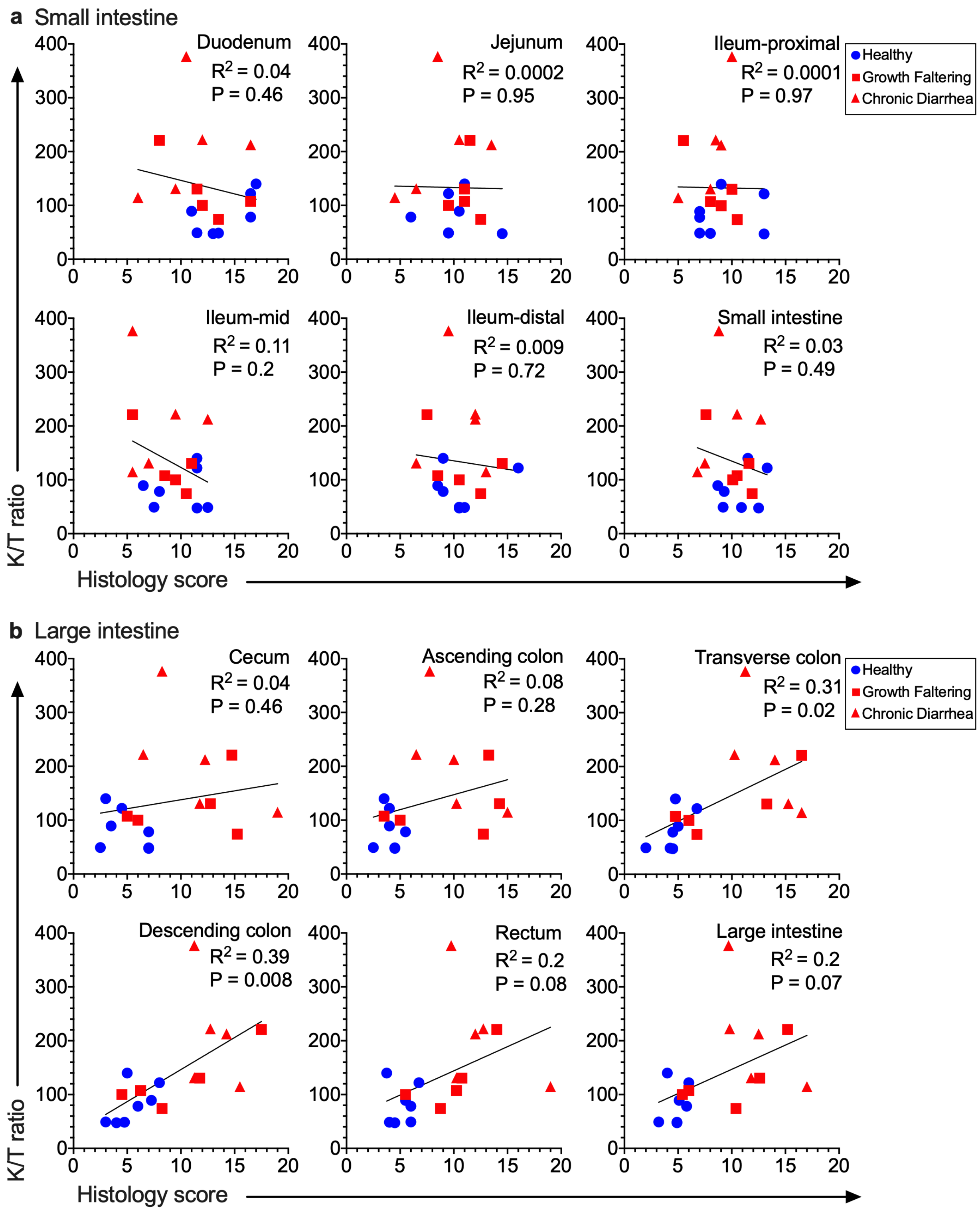

Supplementary Fig. 8. Inclusion of animals with chronic diarrhea in measuring the association between histological scores and K/T ratios (Healthy (n=7, blue circles), Growth Faltering (n=10, red symbols), Chronic diarrhea (n=5, red triangles). P values were determined by univariable linear regression. Source data are provided as a Source Data file.

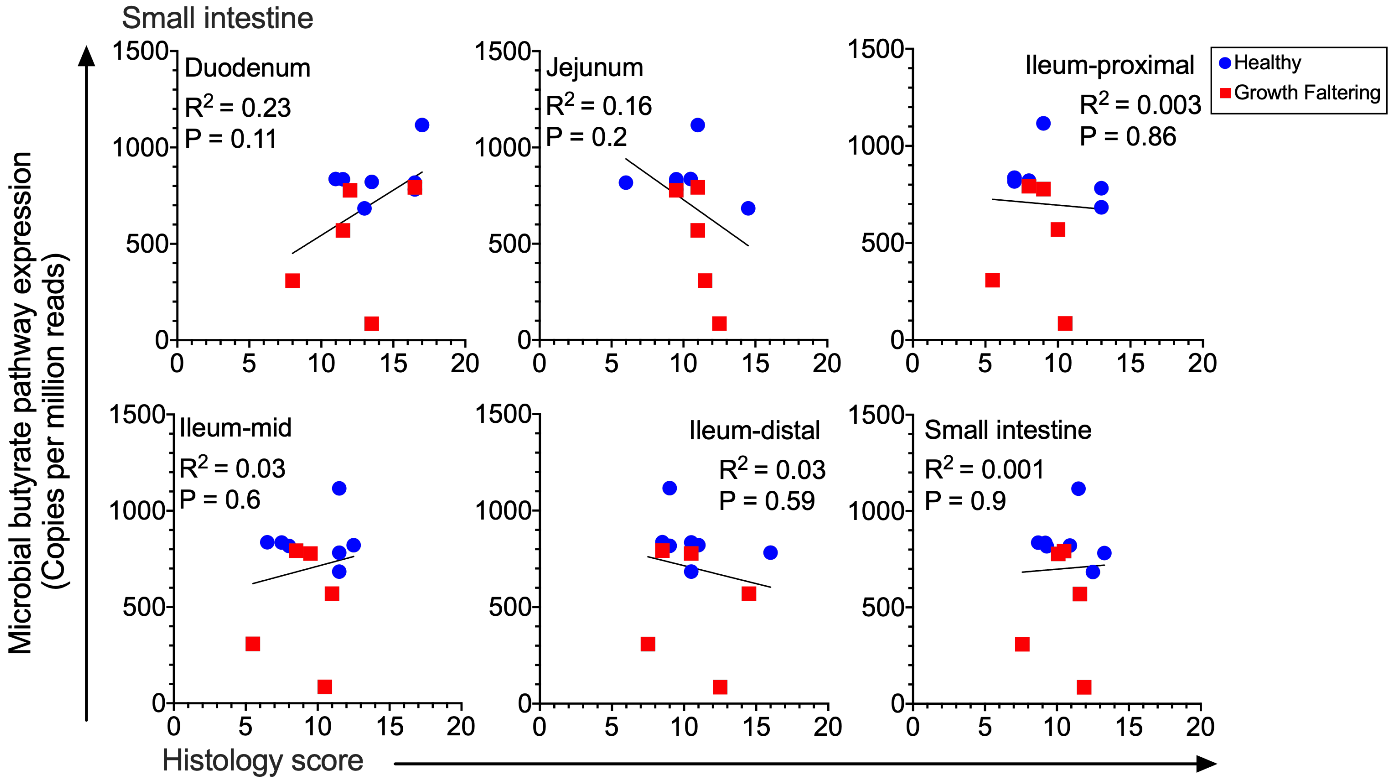

Supplementary Fig. 9. Association between histological scores in the small intestine and colonic microbial butyrate pathway gene expression levels (Healthy (n=7, blue symbols), Growth Faltering (n=5, red symbols)). Animals with chronic diarrhea at the time of necropsy were excluded from analysis. P values were determined by univariable linear regression. Source data are provided as a Source Data file.

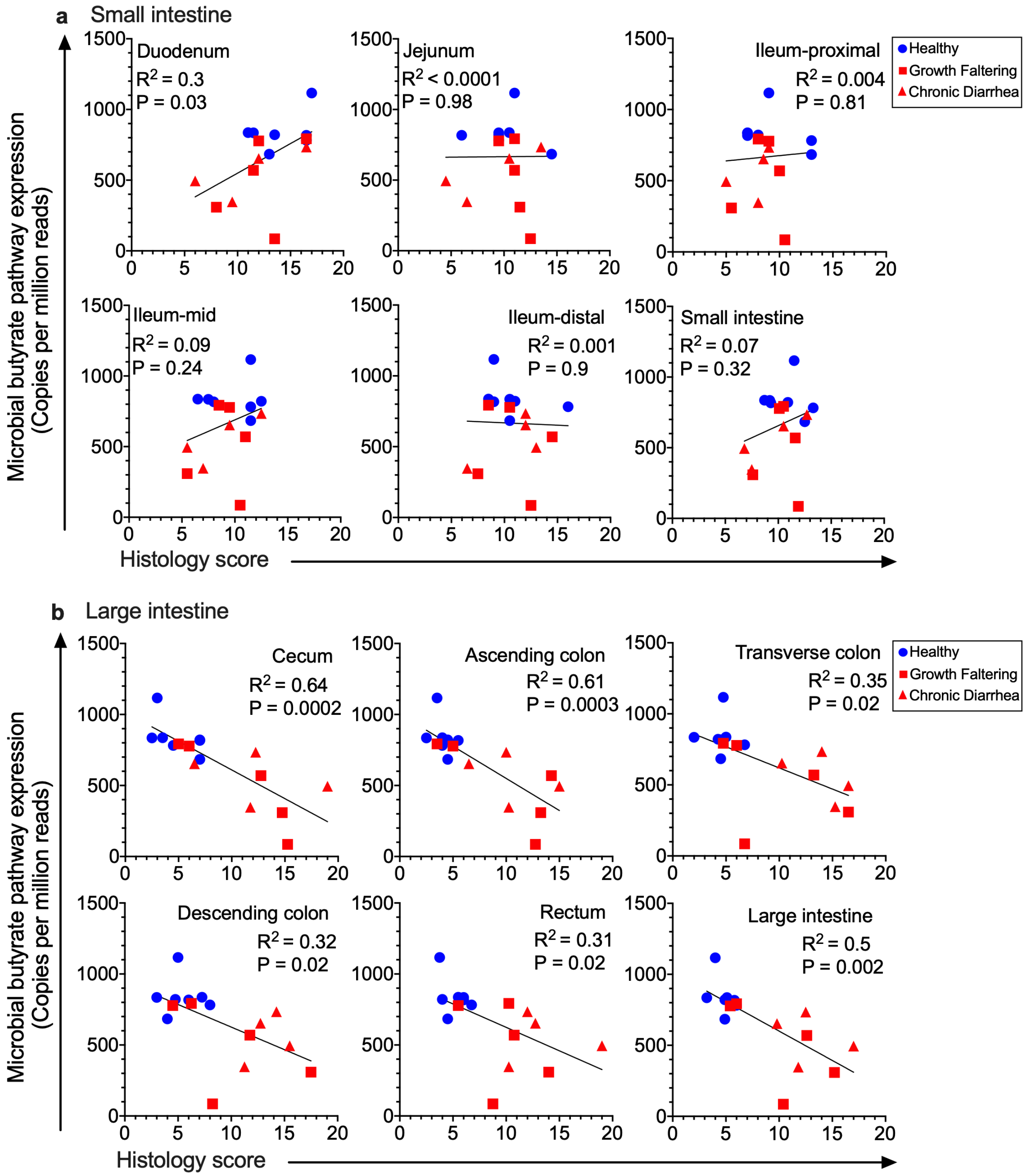

Supplementary Fig. 10. Inclusion of animals with chronic diarrhea in measuring the association between histological scores and colonic microbial butyrate pathway gene expression levels (Healthy (n=7, blue circles), Growth Faltering (n=9, red symbols), Chronic diarrhea (n=4, red triangles). P values were determined by univariable linear regression. Source data are provided as a Source Data file.
