# Supplementary material for: Reduced infant rhesus macaque growth rates due to environmental enteric dysfunction and association with histopathology in the large intestine
Source: Nat Commun. 2022 Jan 11;13:234. doi: 10.1038/s41467-021-27925-x (PMC8752659; doi:10.1038/s41467-021-27925-x)
Supplement: Supplementary file 2 — Reporting summary [file 41467_2021_27925_MOESM2_ESM.pdf]

nature portfolio

Corresponding author(s): Dr.Mark K. Slifka

Last updated by author(s): Nov 29, 2021

Reporting Summary

Nature Portfolio wishes to improve the reproducibility of the work that we publish. This form provides structure for consistency and transparency in reporting. For further information on Nature Portfolio policies, see our Editorial Policies and the Editorial Policy Checklist.

Statistics

For all statistical analyses, confirm that the following items are present in the figure legend, table legend, main text, or Methods section.

n/a	Confirmed	
☐	☒	The exact sample size (*n*) for each experimental group/condition, given as a discrete number and unit of measurement
☒	☐	A statement on whether measurements were taken from distinct samples or whether the same sample was measured repeatedly
☐	☒	The statistical test(s) used AND whether they are one- or two-sided<br>*Only common tests should be described solely by name; describe more complex techniques in the Methods section.*
☒	☐	A description of all covariates tested
☒	☐	A description of any assumptions or corrections, such as tests of normality and adjustment for multiple comparisons
☐	☒	A full description of the statistical parameters including central tendency (e.g. means) or other basic estimates (e.g. regression coefficient) AND variation (e.g. standard deviation) or associated estimates of uncertainty (e.g. confidence intervals)
☐	☒	For null hypothesis testing, the test statistic (e.g. F, t, r) with confidence intervals, effect sizes, degrees of freedom and P value noted<br>*Give P values as exact values whenever suitable.*
☒	☐	For Bayesian analysis, information on the choice of priors and Markov chain Monte Carlo settings
☒	☐	For hierarchical and complex designs, identification of the appropriate level for tests and full reporting of outcomes
☒	☐	Estimates of effect sizes (e.g. Cohen's d, Pearson's r), indicating how they were calculated

Our web collection on statistics for biologists contains articles on many of the points above.

Software and code

Policy information about availability of computer code

Data collection	Shotgun metagenomic libraries were sequenced as 2x100 paired-end reads on an Illumina HiSeq 4000 using HiSeq Control software HD3.4.0. LC-MS/MS data processing and analysis was performed using LabSolutions Software, V5.72 (Shimadzu, Kyoto, Japan)
Data analysis	GraphPad Prism Version 8.2.1(279) was used for student's t-test and CCC analyses were performed in R4.0.4(R Core Team 2021) using DescTools_0.99.41(Signorell 2021) package. Base-calling for Shotgun metagenomics studies was performed using Illumina's RTA version 2.7.7.

For manuscripts utilizing custom algorithms or software that are central to the research but not yet described in published literature, software must be made available to editors and reviewers. We strongly encourage code deposition in a community repository (e.g. GitHub). See the Nature Portfolio guidelines for submitting code & software for further information.

Data

Policy information about availability of data

All manuscripts must include a data availability statement. This statement should provide the following information, where applicable:

- Accession codes, unique identifiers, or web links for publicly available datasets
- A description of any restrictions on data availability
- For clinical datasets or third party data, please ensure that the statement adheres to our policy

The data supporting the results of this study are available within the Source data files provided with this paper. Shotgun metagenomics study reads were functionally annotated using the HUMAnN2 pipeline using default settings with the UniRef50 database. The sequencing datasets used in this study are available in the NCBI SRA repository, under the bioproject ID no. PRJNA729051. Digitized images of the histology slides described in this study have been archived at the Washington University Digital Pathology Exchange (WUPAX).

Field-specific reporting

Please select the one below that is the best fit for your research. If you are not sure, read the appropriate sections before making your selection.

☒ Life sciences ☐ Behavioural & social sciences ☐ Ecological, evolutionary & environmental sciences

For a reference copy of the document with all sections, see nature.com/documents/nr-reporting-summary-flat.pdf

Life sciences study design

All studies must disclose on these points even when the disclosure is negative.

Sample size	Sample size was determined /limited by availability of subjects/samples
Data exclusions	No data was excluded
Replication	Due to some experimental variability, only stool samples that scored positive for a specific enteric pathogen in two independent xTAG experiments were considered positive. Serum tryptophan and kynurenine levels were measured in triplicate. Quantitation of histopathology was performed by two independent/blinded pathologists
Randomization	Histology slides were marked with the specimen type (duodenum, jejunum, ileum, etc) but given randomized identity codes that were not linked to animal group designation or health information for independent evaluation by two pathologists.
Blinding	H&E stained histology slides from different anatomical locations were given a randomized identity code and assessed independently by two blinded pathologists. Analysis of serum trypotphan and kynurenine levels were performed independently by an institutional core laboratory (Endocrine Technologies Support Core) with no information on animal group designation or health history

Reporting for specific materials, systems and methods

We require information from authors about some types of materials, experimental systems and methods used in many studies. Here, indicate whether each material, system or method listed is relevant to your study. If you are not sure if a list item applies to your research, read the appropriate section before selecting a response.

Materials & experimental systems

n/a	Involved in the study	
☐	☒	Antibodies
☒	☐	Eukaryotic cell lines
☒	☐	Palaeontology and archaeology
☐	☒	Animals and other organisms
☒	☐	Human research participants
☒	☐	Clinical data
☒	☐	Dual use research of concern

Methods

n/a	Involved in the study	
☒	☐	ChIP-seq
☒	☐	Flow cytometry
☒	☐	MRI-based neuroimaging

Antibodies

Antibodies used	Mouse monoclonal antibody SMAD7(B-8) sc-365846, Lot E2217 from Santa Cruz Biotechnology, GADPH(6C5) sc-32233 from Santa Cruz Biotechnology
Validation	The SMAD7 antibody was validated by the manufacturer per their associated data sheets described as "Anti-Smad7 Antibody (B-8) is recommended for detection of Smad7 of mouse, rat and human origin by WB, IP, IF and ELISA"

Animals and other organisms

Policy information about studies involving animals; ARRIVE guidelines recommended for reporting animal research

Laboratory animals	Rhesus macaques (Maccacca mulatta), Male and Female, 1 day to 11 months old
Wild animals	The study did not involve wild animals
Field-collected samples	The study did not involve field collected samples

Ethics oversight	The study was performed in strict accordance with the recommendations described in the Guide for the Care and Use of Laboratory Animals of the National Institute of Health, the Office of Animal Welfare and the United States Department of Agriculture. All animal work was approved by the Oregon National Primate Research Center Institutional Animal Care and Use Committee and California National Primate Research Center Institutional Animal Care and Use Committee. Both Centers are accredited by the American Association for Accreditation of Laboratory Animal Care.

Note that full information on the approval of the study protocol must also be provided in the manuscript.
